# Evolution of strigolactone receptors by gradual neo-functionalization of KAI2 paralogues

**DOI:** 10.1186/s12915-017-0397-z

**Published:** 2017-06-29

**Authors:** Rohan Bythell-Douglas, Carl J. Rothfels, Dennis W. D. Stevenson, Sean W. Graham, Gane Ka-Shu Wong, David C. Nelson, Tom Bennett

**Affiliations:** 1 Section of Structural Biology, Department of Medicine, Imperial College London, London, SW7 UK; 2 Integrative Biology, 3040 Valley Life Sciences Building, Berkeley, CA 94720-3140 USA; 3 Molecular Systematics, The New York Botanical Garden, The Bronx, NY USA; 4 Department of Botany, University of British Columbia, 6270 University Boulevard, Vancouver, British Columbia Canada; 5 Department of Medicine, University of Alberta, Edmonton, Alberta Canada; 6 Department of Biological Sciences, University of Alberta, Edmonton, Alberta Canada; 7 BGI-Shenzhen, Beishan Industrial Zone, Yantian District, Shenzhen, China; 8 Department of Botany and Plant Sciences, University of California, Riverside, CA 92521 USA; 9 School of Biology, University of Leeds, Leeds, LS2 9JT UK

**Keywords:** Strigolactone signalling, Strigolactone evolution, Phylogenetics, Neo-functionalization

## Abstract

**Background:**

Strigolactones (SLs) are a class of plant hormones that control many aspects of plant growth. The SL signalling mechanism is homologous to that of karrikins (KARs), smoke-derived compounds that stimulate seed germination. In angiosperms, the SL receptor is an α/β-hydrolase known as DWARF14 (D14); its close homologue, KARRIKIN INSENSITIVE2 (KAI2), functions as a KAR receptor and likely recognizes an uncharacterized, endogenous signal (‘KL’). Previous phylogenetic analyses have suggested that the KAI2 lineage is ancestral in land plants, and that canonical D14-type SL receptors only arose in seed plants; this is paradoxical, however, as non-vascular plants synthesize and respond to SLs.

**Results:**

We have used a combination of phylogenetic and structural approaches to re-assess the evolution of the D14/KAI2 family in land plants. We analysed 339 members of the D14/KAI2 family from land plants and charophyte algae. Our phylogenetic analyses show that the divergence between the eu-KAI2 lineage and the DDK (D14/DLK2/KAI2) lineage that includes D14 occurred very early in land plant evolution. We show that eu-KAI2 proteins are highly conserved, and have unique features not found in DDK proteins. Conversely, we show that DDK proteins show considerable sequence and structural variation to each other, and lack clearly definable characteristics. We use homology modelling to show that the earliest members of the DDK lineage structurally resemble KAI2 and that SL receptors in non-seed plants likely do not have D14-like structure. We also show that certain groups of DDK proteins lack the otherwise conserved MORE AXILLARY GROWTH2 (MAX2) interface, and may thus function independently of MAX2, which we show is highly conserved throughout land plant evolution.

**Conclusions:**

Our results suggest that D14-like structure is not required for SL perception, and that SL perception has relatively relaxed structural requirements compared to KAI2-mediated signalling. We suggest that SL perception gradually evolved by neo-functionalization within the DDK lineage, and that the transition from KAI2-like to D14-like protein may have been driven by interactions with protein partners, rather than being required for SL perception per se.

**Electronic supplementary material:**

The online version of this article (doi:10.1186/s12915-017-0397-z) contains supplementary material, which is available to authorized users.

## Background

Plant hormones are a key link between environmental stimuli and development, allowing local information to be used systemically across the plant body. Strigolactones (SLs) are a recently identified class of terpenoid lactone hormones that neatly epitomize this concept. SLs are primarily synthesized by a core pathway involving a carotene isomerase (DWARF27), two carotenoid cleavage dioxygenases (CCD7 and CCD8) [1] and a cytochrome P450 enzyme (MAX1). SL synthesis is strongly upregulated by phosphate deficiency in the rhizosphere [2], increasing the pool of SL molecules in the root. In many flowering plants (angiosperms), SLs are exuded into the soil through the action of specific SL transporters and serve to attract mycorrhizal fungi [3]; the resulting symbioses provide the plants with phosphate in exchange for reduced carbon. SLs also act locally to regulate root system architecture; the precise effects seem to vary from species to species, but increased SL levels may promote increased nutrient foraging [4]. Finally, a significant proportion of the SL pool produced in the root is transported into the shoot system via the xylem [5], where it has a well-defined set of effects on shoot growth and development [6, 7]. SL has an inhibitory effect on shoot branching, thereby coupling shoot growth to nutrient availability [5]. SL responses thus form an integrated stimulus-response system acting over long distances both within the plant body and in its immediate environment.

Like several other plant hormonal signalling pathways, canonical SL signalling is mediated through ubiquitin-mediated degradation of target proteins (reviewed in [7]). The SL receptors for this signalling pathway are members of the DWARF14 (D14) class of α/β-hydrolase proteins, which are an unusual combination of enzyme and receptor [8, 9]. D14 proteins bind and then cleave SL molecules, producing a covalently linked intermediate molecule (CLIM) that is covalently bound to the receptor [8, 9]. SL signalling is mediated through the interaction of D14 with the MORE AXILLARY GROWTH2 (MAX2) class of F-box proteins, which forms part of an SCF (SKP1-CULLIN-F-BOX) E3 ubiquitin ligase [10–13]. Together, the covalent binding of CLIM and the interaction with SCF^MAX2^ allow D14 to undergo a stable conformational change that drives onward signalling [8, 9]. Although other targets have been proposed [14, 15], it is now clear that the principal proteolytic targets of SL signalling are proteins of the SMAX1-LIKE7/DWARF53 (SMXL7/D53) class [16–21]. The exact sequence of events is unclear, but it is probably after conformational change that D14 stably recruits SMXL7 to the complex; certainly, the D14-SMXL7 interaction is enhanced by SL [16, 17, 19, 20]. Events downstream of SMXL7 degradation are currently poorly defined; SMXL7 has been proposed to act both transcriptionally and non-transcriptionally [7, 22]. It may be that SMXL7 is a multi-functional protein that can regulate multiple cellular processes [20].

Intriguingly, a second pathway in angiosperms signals through SCF^MAX2^, forming a biochemical and evolutionary parallel to SL signalling. This pathway is defined by the KARRIKIN INSENSITIVE2 (KAI2) α/β-hydrolase protein, a close relative of D14. *kai2* mutants have a range of developmental phenotypes [18, 21, 23] and are insensitive to the germination-promoting effects of smoke-derived ‘karrikins’ (KARs) [23]. It has been hypothesized that karrikins promote germination by mimicking an as-yet-unidentified endogenous KAI2 ligand (‘KL’) [24, 25]. The KAI2 orthologue in rice (D14-LIKE) is also required for the establishment of mycorrhizal associations in the root system [26]. It is currently unclear whether D14-LIKE perceives a fungal signal or endogenous KL in this context. As with D14, MAX2 (and orthologues) is required for both responses to karrikins and for other aspects of KAI2-dependent signalling [18, 21, 27]. Furthermore, the presumptive proteolytic targets of KAI2-SCF^MAX2^ signalling are close homologues of SMXL7; in Arabidopsis, these are SMXL2 and SMAX1 (SUPPRESSOR OF MAX2 1). Mutation of *SMAX1* and *SMXL2* suppresses the *kai2*-related phenotypes present in the *max2* mutant, producing phenotypes that mimic constitutive karrikin responses [18, 28, 29]. In the Arabidopsis genome, there are further homologues of *D14* and *SMAX1*, namely *DWARF14-LIKE2* (*DLK2*) and *SMXL3, SMXL4* and *SMXL5*, but the function of these proteins and their relationship to SL/KL signalling is currently unclear [23, 28].

The evolutionary history of SLs represents an intriguing and unresolved problem. SLs have been identified in most land plant groups, and in some related groups of charophyte algae [30]. However, unambiguous *CCD8* orthologues have not been identified in charophytes or liverworts [30] (a possible sister group to other land plants [31]). Moreover, *ccd8* mutants in the moss *Physcomitrella patens* still produce some SLs [32], which suggests that there may be alternative pathways for SL synthesis [7, 33]. Even more uncertainty surrounds the origin of the canonical SL signalling pathway. Unambiguous *D14* orthologues have only been identified in seed plants (gymnosperms and angiosperms), and they seem to be absent from mosses and liverworts [30, 34]. Conversely, it has been suggested that unambiguous *KAI2* orthologues are present in charophytes, liverworts and mosses [30]. This has led to the suggestion that KAI2 proteins could function as receptors for SLs in non-vascular plants, or that SL signalling occurs by non-canonical mechanisms in these lineages [7, 22]. Supporting the plausibility of the former hypothesis, it was recently shown that SL receptors evolved from *KAI2* paralogues in parasitic plants within the Orobanchaceae [35–37]. In addition, *MAX2* orthologues have so far only been identified in land plants [38], and while *MAX2* is present in *P. patens*, *Ppmax2* mutants do not resemble *Ppccd8* mutants, suggesting that MAX2 may not be involved in SL signalling in mosses [22, 39]. Thus, even if KAI2 proteins can act as SL receptors in mosses, they may not act through SCF^MAX2^-mediated protein degradation. SMXL proteins are present in *P. patens*, but their function has not been investigated. Thus, while there is clear evidence for SL sensitivity in mosses, it is possible that this occurs through separate mechanisms to those in angiosperms. This would contrast strongly with the auxin signalling pathway for instance, which is completely conserved throughout land plants [40–42]. To resolve the evolutionary history of SL signalling, we have undertaken a major phylogenetic re-assessment of the *D14*/*KAI2* family.

## Results

### Preliminary analysis of the *D14*/*KAI2* family

In order to understand the evolution of the *D14*/*KAI2* family with greater resolution, we obtained 339 sequences from 143 species, representing the major lineages of land plants and charophyte algae (summarized in Additional file 1). All preliminary phylogenetic analyses placed *D14*/*KAI2* family members into unambiguous taxon-specific clades such as angiosperm *KAI2* or gymnosperm *D14* (Table 1). Understanding the interrelationship of these taxon-level clades therefore seemed to be key to understanding the evolution of the *D14*/*KAI2* family. Sequences from each major land plant taxon grouped into at least two distinct clades, except for the hornworts, in which all sequences grouped into a single clade (Table 1).

**Table 1:** Major clades in the *D14*/*KAI2* family

	Clade	Taxon	Sequences	Major sub-clades
	*KAI2*	Klebsormidiales	2	
	*KAI2*	Charales	1	
	*KAI2*	Coleochaetales	3	
	*KAI2*	Zygnematales	4	
	*NK2*	Zygnematales	5	
*Eu-KAI2*	*KAI2A*	Liverworts	9	
	*KAI2C/D*	Mosses	19	*KAI2C, KAI2D*
	*KAI2*	Hornworts	5	
	*KAI2*	Lycophytes	15	
	*KAI2*	Monilophytes	27	*KAI2G, KAI2H*
	*KAI2*	Gymnosperms	18	*KAI2I, KAI2J*
	*KAI2*	Angiosperms	34	
*DDK*	*KAI2B*	Liverworts	6	
	*KAI2E/F*	Mosses	11	*KAI2E, KAI2F*
	*DDK*	Lycophytes	7	
	*DDK*	Monilophytes	15	*DDKA, DDKB*
	*DLK4*	Gymnosperms	18	*DLK4A, DLK4B*
	*D14*	Gymnosperms	10	
	*D14*	Angiosperms	37	
	*DLK23*	Gymnosperms	23	
	*DLK23*	Angiosperms	70	*DLK2, DLK3*

Table showing major clades in the *D14*/*KAI2* family, as defined at the level of major taxonomic groups. Almost all sequences in the family unambiguously group into one of these clades. Within some clades there are major sub-clades where the lineage has been duplicated; these are listed at the right. Our analysis suggests that land plant *D14*/*KAI2* proteins group into two super-clades, *eu-KAI2* and *DDK*, as indicated on the *left* of the table

**Fig. 1:**
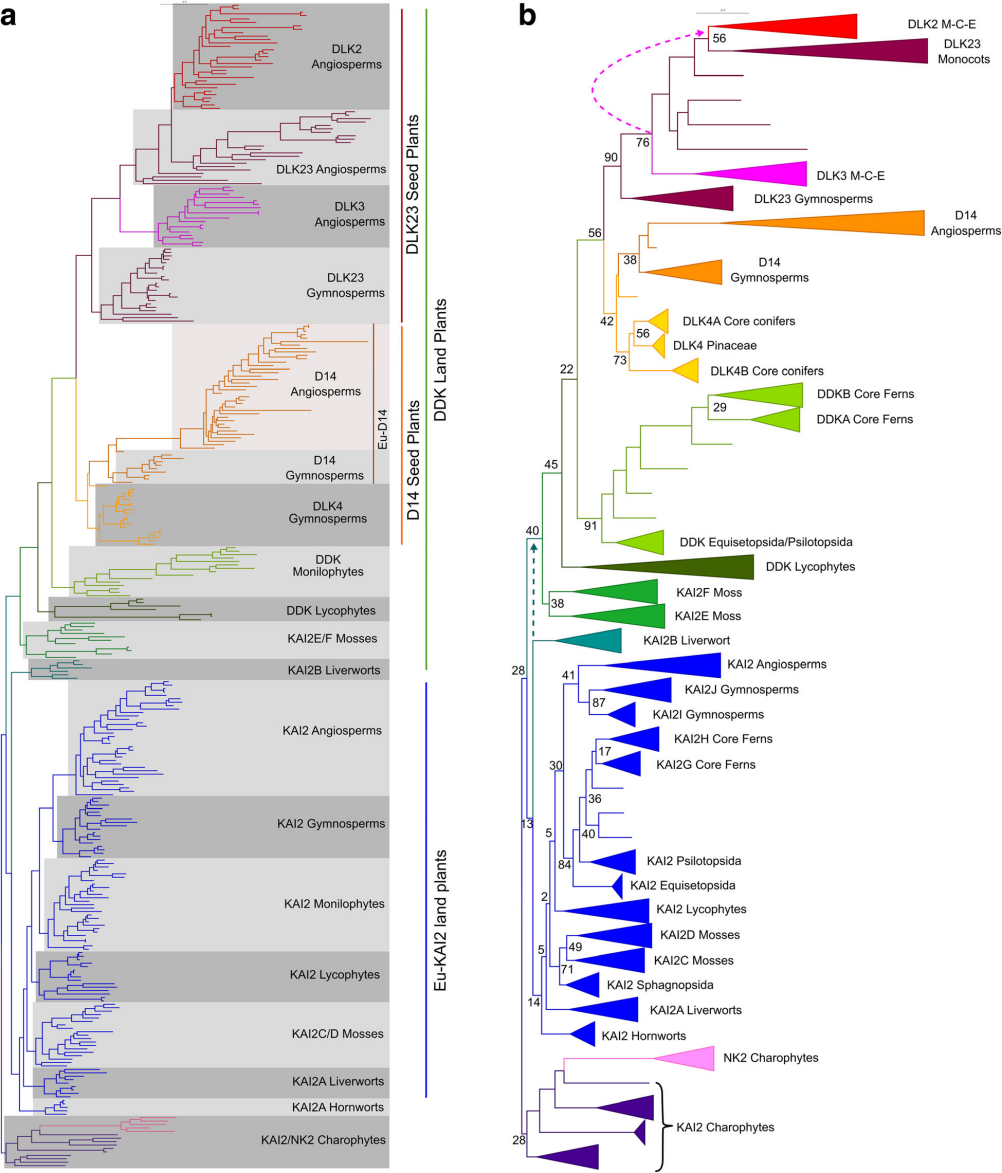
The *eu-KAI2* and *DDK* super-clades diverged early in land plant evolution. Codon-level phylogenetic analysis implemented in the Genetic Algorithm for Rapid Likelihood Inference (*GARLI*) on the whole *D14*/*KAI2* family (339 sequences from 143 species). This analysis was performed using an optimized character set (see Methods). Trees were rooted with charophyte sequences, consistent with contemporary notions of plant organismal phylogeny. *Dotted lines* indicate alternative positions for the indicated clades that would increase the parsimony of the tree. **a** Phylogram showing the ‘most likely’ tree from GARLI analysis, labelled to show the high-order relationships between the major clades (as described in Table 1). **b** Cladogram depicting the phylogenetic tree from (**a**) in simplified form. Major clades and sub-clades (as listed in Table 1) are collapsed. Numbers associated with internal branches denote maximum likelihood bootstrap support (percent support). *M*-*C*-*E* magnoliids-chloranthales-eudicots

From species in the charophyte orders Klebsormidiales, Charales and Coleochaetales we only obtained a single sequence per genome, all of which superficially resembled *KAI2*. However, from several species in the Zygnematales we obtained two distinct types of sequences, one resembling *KAI2* and the other not, which we named *NOT KAI2* (*NK2*). Reciprocal Basic Local Alignment Search Tool (BLAST) searches did not identify any NK2-like sequence in complete chlorophyte alagal genomes, or any NK2-like sequences in other charophyte or embryophyte transcriptomes/genomes, except for known *D14*/*KAI2*/*DLK2* sequences. In recent analyses the Zygnematales have been identified as good candidates for the sister group to land plants, even though morphological analyses have traditionally favoured the Charales in this respect [31, 43–45]. If this reconstruction is correct, the two lineages present in Zygnematales could be evidence that the duplication in the *D14*/*KAI2* family occurred before the land plant-Zygnematales split. However, in our analyses *NK2* sequences grouped with other charophyte *KAI2* sequences (Fig. 1), and they have highly divergent characteristics, unlike any other members of the *D14*/*KAI2* family. The available evidence thus suggests that these genes are unique to the Zygnematales and arose from a gene duplication event within that lineage.

### Multiple analyses support an early origin for the DDK super-clade

**Fig. 2:**
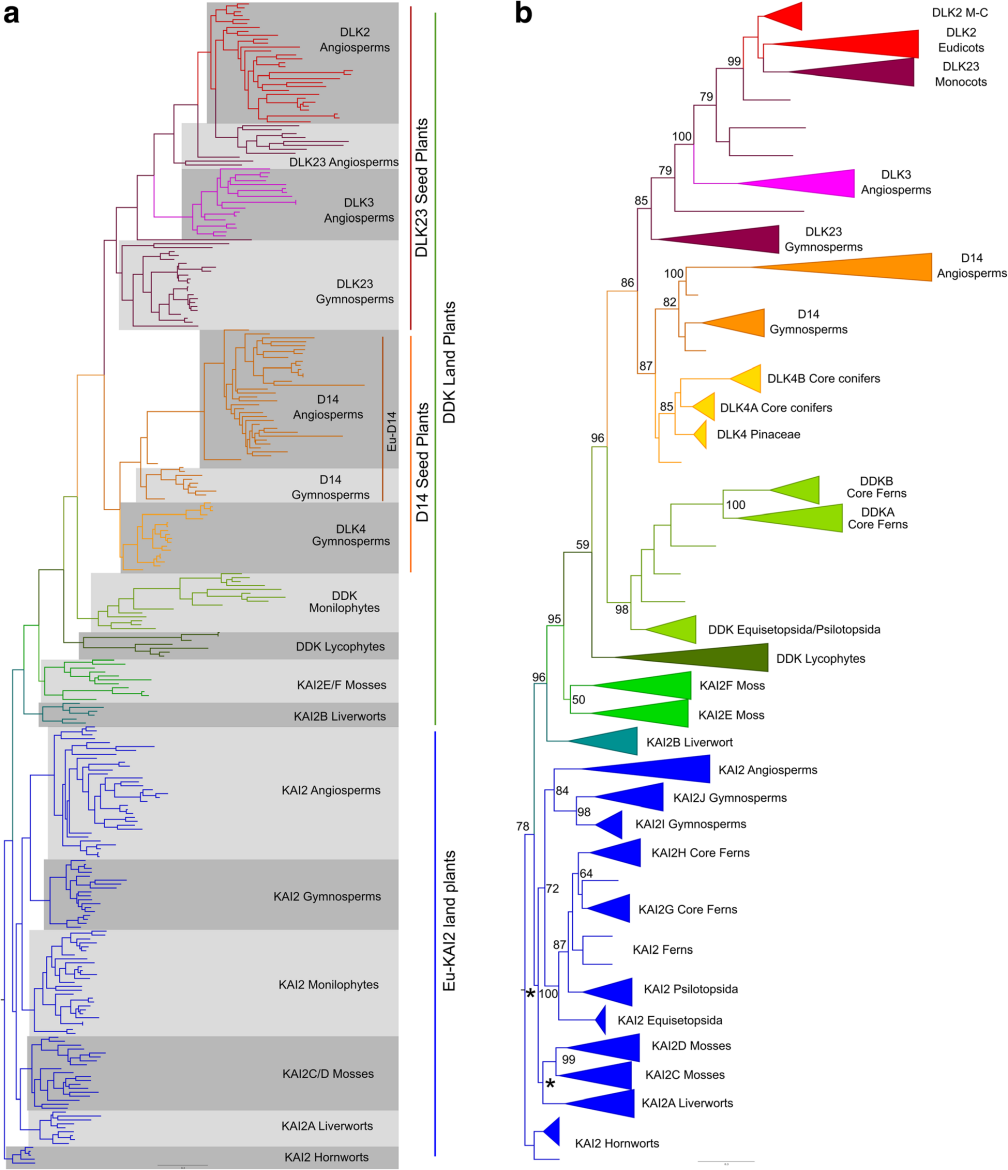
The *eu-KAI2* and *DDK* super-clades diverged early in land plant evolution. Nucleotide-level phylogenetic analysis implemented in GARLI on the *D14*/*KAI2* family, minus charophyte and lycophyte *KAI2* sequences (296 sequences). Trees were rooted with hornwort *KAI2* sequences by comparison with Fig. 1. This analysis was performed using the full-length dataset (780 characters). **a** Phylogram showing the ‘most likely’ tree from GARLI analysis, labelled to show the high-order relationships between the major clades (as described in Table 1). **b** Cladogram depicting the phylogenetic tree from (**a**) in simplified form. Major clades and sub-clades (as listed in Table 1) are collapsed. Numbers associated with internal branches denote maximum likelihood bootstrap support (percent support); values below 50 are indicated by *. *M*-*C* magnoliids/chloranthales

To explore the evolution of the *D14*/*KAI2* family, we performed maximum likelihood phylogenetic analyses using both nucleotide and amino acid sequence data, implemented in PhyML and Genetic Algorithm for Rapid Likelihood Inference (GARLI) [46, 47]. Preliminary analyses were run on a ‘maximum’ alignment of 780 nucleotides from all 339 sequences, and the resulting trees rooted with charophyte sequences. However, we found that lycophyte *KAI2* sequences (particularly those from *Selaginella* spp.) tended to be misplaced near the root of the tree. This is a recognized problem in land plant phylogenies, caused by divergent codon usage in lycophytes (particularly *Selaginella*), which resembles that of charophytes [48]. We were able to improve the overall tree topology, resulting in more realistic branching orders, by using progressively smaller and more conservative alignments (Fig. 1, Additional file 2). If we removed the charophyte and lycophyte sequences (leaving 296 sequences from 122 species), we were able to recover the same basic topology, but using the maximum DNA alignment (Fig. 2, Additional file 3).

Irrespective of the underlying alignment and methodology, all analyses agreed on a basic topology for the family, with a deep duplication near the base of the land plants creating two super-clades. The first lineage contains *KAI2* sequences from angiosperms and closely related sequences from gymnosperms, monilophytes, lycophytes, mosses and liverworts; we therefore named this clade *eu-KAI2* (Table 1). The second super-clade contains sequences from mosses that have previously been described as *KAI2*-like [23, 34, 49], sequences from lycophytes and monilophytes that do not resemble known proteins, the previously characterized *D14* and *DLK2* genes from angiosperms and homologous genes from gymnosperms (Table 1). To reflect the mixed composition of this clade, we named it ‘*DDK*’ (for *D14*/*DLK2*/*KAI2*); we also used this name for the monilophyte and lycophyte sequences in the clade. The lycophyte *DDK* group contains the *Selaginella moellendorffii* gene previously described as ‘*SmKAI2b*’ [34], but we believe *DDK* designation better reflects the evolutionary context of these proteins. We observed some variation in the composition of the *eu-KAI2* clade, partly as a result of the erratic behavior of the lycophyte *KAI2* sequences. However, the moss *KAI2E*/*F*, lycophyte *DDK*, monilophyte *DDK*, gymnosperm *D14*, *DLK4* and *DLK23* and angiosperm *D14* and *DLK23* clades were associated into a single large clade in every analysis we performed, although the internal branching order did vary somewhat between analyses. This basic topology was evident even in very early analyses (Additional file 4).

Only two clades were inconsistently placed. The hornwort *KAI2* clade is the most problematic in our analyses, mirroring the uncertainty about the position of the hornworts themselves in organismal phylogeny [31]. In some analyses the hornwort *KAI2* clade is placed in the *eu-KAI2* lineage, between mosses and vascular plants (Additional file 3). Alternatively, it is also placed at the base of the *eu-KAI2* lineage (Fig. 1) or as a sister clade to all other land plant *D14*/*KAI2* sequences (Fig. 2, Additional file 2). None of these positions alter the interpretation of a deep duplication in the family, but they do affect its inferred timing. The liverwort *KAI2B* clade occurs either at the base of the *DDK* or *eu-KAI2* lineages in different trees. In analyses performed without charophyte and lycophyte *KAI2* sequences, it is always associated with the *DDK* lineage (Fig. 2, Additional file 3). This is also the case in some analyses including charophyte sequences (Additional file 2). The position at the base of the *eu-KAI2* clade in some trees is likely to be erroneous and is probably caused by the slight misplacement of charophyte sequences. For instance, the liverwort-hornwort-liverwort branching order at the base of the *eu-KAI2* clade in Fig. 1 is highly improbable. Rooting this tree with the hornwort *KAI2* clade (to match Fig. 2) produces balanced *eu-KAI2* and *DDK* clades, with realistic branching order, except for the inclusion of the charophyte sequences as an in-group within the *DDK* clade (Additional file 5). We believe the most parsimonious scenario is that *KAI2B* is part of the *DDK* clade.

Collectively, our phylogenetic analyses push the origin of the *D14* lineage back much earlier than proposed in previous phylogenies that suggested an origin in the vascular plants [23] or within the seed plants [34]. They resolve the enigmatic placement of *SmKAI2b* and divergent *KAI2* sequences from *P. patens* in previous phylogenies [34, 49]. They also provide a convincing explanation for the presence of two distinct *D14*/*KAI2* clades in most major plant groups. Key to this reconstruction topology is the placement of liverwort and moss clades with apparently KAI2-like primary protein structure (KAI2B and KAI2E/F respectively) in the *DDK* lineage. We wanted to test the robustness of this somewhat unexpected conclusion, and used a variety of methods to do so.

Non-parametric bootstrap analyses performed in GARLI did not provide very high levels of support for most of the nodes along the backbone of the tree (Fig. 1). However, bootstrap values were higher in reconstructions that excluded charophyte and lycophyte *KAI2* sequences (Fig. 2). We next tested whether the recovered topology was stable to perturbations in the dataset. We re-ran our analysis multiple times, removing each *DDK* clade in turn (see Methods). Our analysis suggests that the placement of *KAI2B* is sensitive to the dataset used, but that the rest of the *DDK* clade is very stably associated (Additional file 6). Finally, we assessed whether our general topology is congruent with previous analyses. We observed that in [23], the *Marchantia polymorpha KAI2A* and *KAI2B* sequences do not group together, and neither do the *P. patens KAI2C*/*D* and *KAI2E*/*F*. This is consistent with our analyses. We repeated our analysis using a set of sequences pruned to match [23] and found essentially the same tree as in that study (Additional file 6). Furthermore, if we rooted the tree with a *eu-KAI2* sequence, we observed essentially the same topology as in our study (Additional file 7). This shows that the difference in final topology between our study and [23] does not result from any particular methodological differences, but from our more densely populated sequence set.

### Diverse evolutionary histories in the *D14*/*KAI2* family

**Fig. 3:**
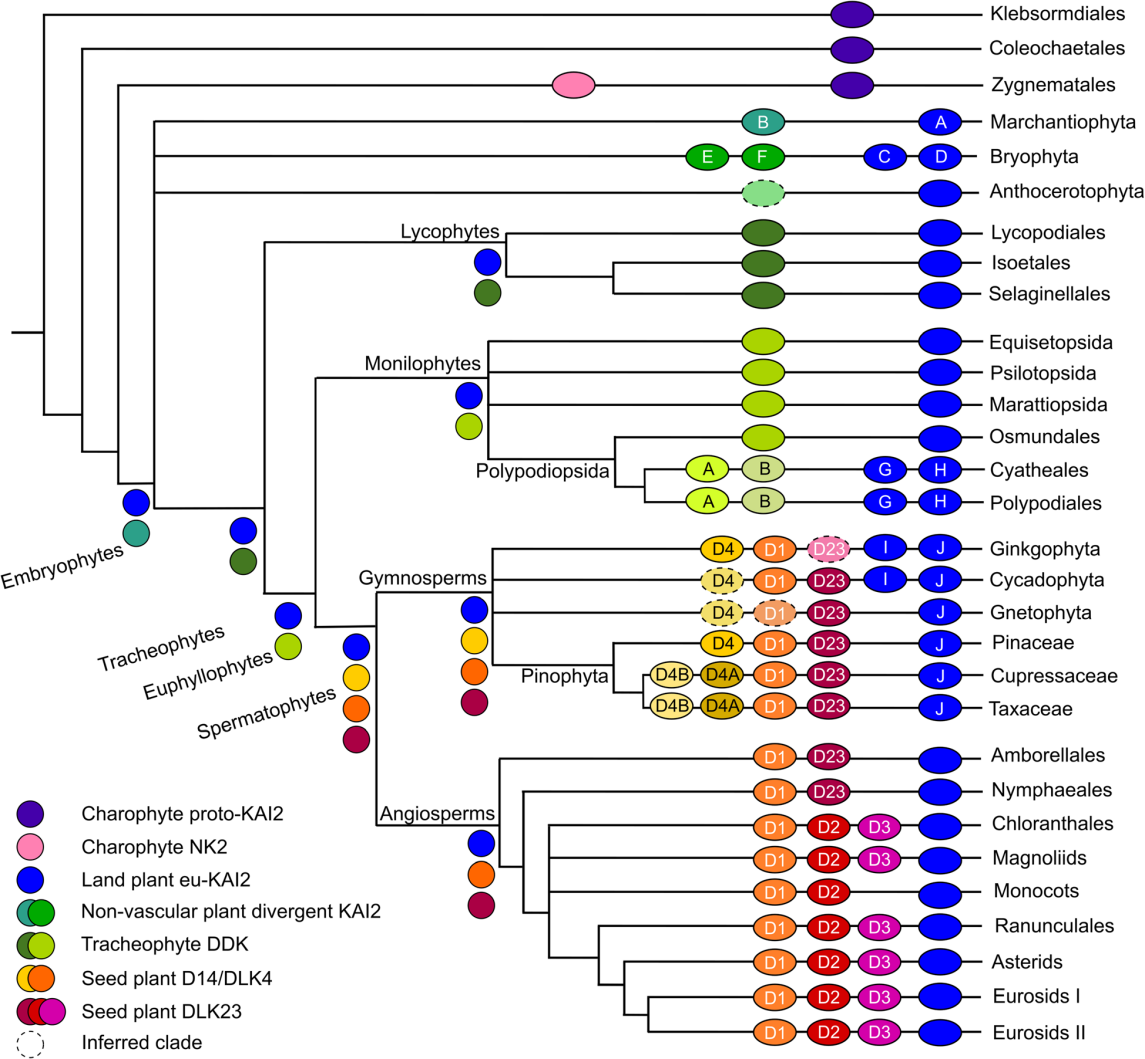
Reconstruction of D14/KAI2 family evolution. Schematic depicting the complement of D14/KAI2 proteins in major land groups, and their inferred evolutionary origin. Each branch indicates a major land plant group; lycophytes, monilophytes and gymnosperms are further sub-divided into relevant orders/families/etc. The *ovals* on each branch indicate the core complement of proteins in that group or sub-group and are coloured according to the scheme indicated at the *bottom left*. Clades which are inferred by parsimony are denoted with a *hatched line. Letters and numbers in the ovals* denote the clade names as outlined in Table 1. *Letters and numbers in the circles* indicate clade names. D1 = D14, D2 = DLK2, D3 = DLK3, D4 = DLK4, D23 = DLK23. *Circles without symbols* at internal branching points represent the minimum inferred D14/KAI2 protein complement in the last common ancestor of each major land plant group

**Fig. 4:**
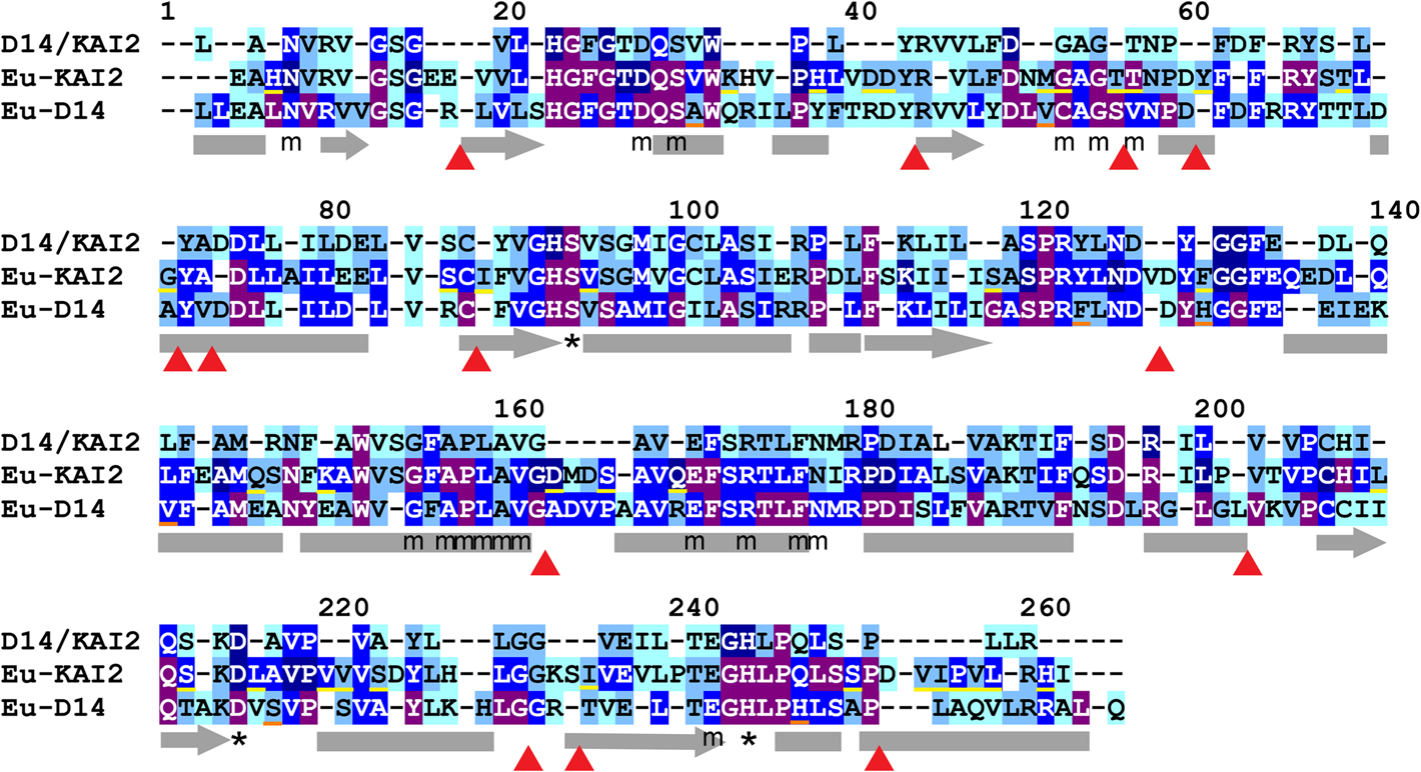
Eu-KAI2 proteins have highly conserved structure. Alignment illustrating conservation of primary protein structure in D14/KAI2 proteins. The 265 core positions (*numbered*) are shown in the alignment, for the whole family (*top row*), for eu-KAI2 proteins (*middle row*) and for eu-D14 proteins (*bottom row*). Positions where the same amino acid is present in >50% of sequences in the clade are denoted by *corresponding letter*; other positions are denoted by a *dash*. The colouring of each conserved residue indicates the degree of conservation; *pale blue* >50%, *light blue* >70%, *mid-blue* >90%, *dark blue* >99%, *purple* =100%. Structural features are annotated below the alignment. The catalytic triad is indicated by *. MAX2-interacting residues are indicated by *m*. Predicted alpha helices (based on the crystal structure of AtKAI2 (Protein Data Bank (*PDB*) code 4HRX1A) are shown by *grey bars*, predicted beta strands by *grey bars with an arrow*. The discrete positions in the polypeptide chain where insertions (or deletions) can be tolerated are illustrated with *red arrow heads*. Residues that are characteristic of eu-KAI2 proteins are underlined in *yellow*; residues characteristic of eu-D14 are underlined in *orange* (see Fig. 5)

From our phylogenetic reconstruction, it is apparent that the two super-clades of the *D14*/*KAI2* family appear to have rather different evolutionary trajectories (Fig. 3). Within the *eu-KAI2* super-clade there is a single clade for each major plant group (e.g. angiosperm *KAI2*). Within these taxon-specific clades, there have apparently been some early duplications. For instance, *KAI2C* and *KAI2D* clades are widely represented among extant mosses, although not in the Sphagnopsida, suggesting that the duplication occurred after the separation of the Sphagnopsida and other mosses (Fig. 1). Similarly, the separation of the *KAI2I* and *KAI2J* clades must have occurred relatively early in gymnosperm evolution, since both proteins are found in ginkgo and cycads, although *KAI2I* is not found in conifers (Figs. 3 and 4). There are also many local duplications in the *KAI2* lineage, with some species having up to five *eu-KAI2* paralogues. However, the overall evolutionary trend in the *eu-KAI2* clade (as also suggested by the generally short branch lengths) is one of conservation rather than innovation (Figs. 1 and 3).

Conversely, the evolutionary history of the *DDK* clade is one of divergence and diversification. The liverwort and moss clades (*KAI2B*, *KAI2E*/*F*) are on relatively short branches (Fig. 3) and have been categorized previously as encoding KAI2-like proteins. The lycophyte and monilophyte ‘DDK’ proteins are neither obviously similar to the previously described KAI2, D14 or DLK2 protein types, nor indeed to each other. These clades also have long internal branch lengths, indicating a high degree of sequence divergence within the clades (Fig. 3). In the leptosporangiate fern core group there has been a duplication in the *DDK* lineage, and the resulting DDKA and DDKB protein types are strongly divergent both from each other and from other monilophyte DDK proteins. In seed plants, there are a number of major duplications and evidence for significant innovation in protein sequence (Fig. 3). In gymnosperms, we identified *eu-D14* sequences that form a sister clade to the well-characterized angiosperm *D14* clade. We also identified a second set of sequences in gymnosperms that are closely related to *D14*, which we named *DWARF14-LIKE4* (*DLK4*). These form a sister clade to the gymnosperm/angiosperm *eu-D14* clade, suggesting that the duplication that gave rise to *DLK4* occurred before the separation of gymnosperms and angiosperms (Fig. 1). This in turn implies that the *DLK4* clade has been lost from angiosperms (Fig. 3). Within the conifers there has been a major duplication in the *DLK4* lineage giving rise to two sub-clades (*DLK4A* and *DLK4B*); since *DLK4B* is not found in Pinaceae, the separation of *DLK4A* and *DLK4B* seems to post-date the divergence of pines and other conifers (Fig. 3).

In angiosperms, we also discovered a third clade of proteins in addition to the expected *D14* and *DLK2* clades, which appeared as a sister clade to *DLK2* in our analysis; we named these sequences *DWARF14-LIKE3* (*DLK3*) (Fig. 1). Although our phylogenetic reconstruction suggests that the separation of *DLK2* and *DLK3* occurred before the radiation of extant angiosperms, the distribution of *DLK3* sequences in our dataset suggests a slightly different history. We did not recover any *DLK3*-like sequences from the completed genome sequence of *Amborella trichopoda* (the sister group to all other angiosperms) or from the plants in the other early-diverging angiosperm orders (Nymphaeales, Austrobaileyales). We did identify unambiguous *DLK3* sequences from the Chloroanthales and magnoliids, but not from any monocot species (including the fully sequenced genomes in Poaceae), despite extensive screening; we could however identify *DLK2* sequences from across the monocot group. *DLK3* sequences are present throughout the eudicots, although there have been sporadic losses, including some in the Brassicaceae. The exact inter-relationship of the major angiosperm lineages is currently uncertain, but one well-supported model is that monocots are sister to a clade containing magnoliids, Chloranthales and eudicots [31]. Under this scenario, the distribution of genes suggests that the separation of the *DLK2* and *DLK3* lineages occurred after the divergence of monocots and other angiosperms (Fig. 3). Alternatively, *DLK3* could have been lost from the monocot lineage. We also identified a group of gymnosperm proteins that form a sister group to the combined angiosperm *DLK2*-*DLK3* clade, which we named *DLK23*. We also applied this name to the angiosperm proteins that pre-date the *DLK2*-*DLK3* split, and to the wider seed plant clade containing all these proteins (Figs. 1 and 3).

### Sequence conservation among D14/KAI2 proteins

To further understand the consequences of the evolutionary trajectories of the *D14*/*KAI2* family members, we performed an in-depth analysis of their primary protein structure. Using our alignment, we identified a core set of 265 positions that occur in almost every D14/KAI2 protein (Fig. 4). The start and end positions of the polypeptide chain vary between individual sequences, but the majority of sequences are within the range –15 to 280. Extra amino acids are inserted within the core of the protein in some sequences; these are usually located outside secondary structural elements such as α-helices (Fig. 4). Most of these insertions are not conserved even between closely related sequences, although there are some exceptions. For instance, DDKB proteins from monilophytes have a conserved insertion of five amino acids after position 73.

In order to make comparisons across the family, we focussed our attention on the core positions 1–265. We examined the amino acid frequency at each of these core positions, in different sub-sets of sequences, and used the data to understand patterns of conservation and divergence. We classify a position as ‘conserved’ if the same amino acid occurs in more than 50% of sequences in the sub-set, ‘well conserved’ if found in more than 70% of sequences, ‘highly conserved’ if found in more than 90% of sequences and ‘invariant’ if found in more than 99% of sequences. Using this methodology on the D14/KAI2 family as a whole (339 sequences), we found that 68% of positions are conserved, with 18.5% being highly conserved (Fig. 4). Of these, 17 positions (6.4%) are invariant, including the catalytic triad of serine, aspartate and histidine (positions 94, 215, 244 respectively) (Fig. 4, Table 2). Most of the highly conserved residues cluster together in the polypeptide chain, forming motifs that are presumably important for protein activity (Fig. 4).

**Table 2:** Protein sequence conservation in D14/KAI2 proteins

	Invariant	Highly	Well	Conserved
Whole family	6.8	17.7	42.6	68.3
Eu-KAI2	22.3	50.6	**72.5**	**89.1**
Lycophyte KAI2	48.3	60.4	**83.0**	**95.1**
Angiosperm KAI2	24.5	54.7	**76.2**	**94.3**
DDK super-clade	5.7	17.7	34.0	63.8
Gymnosperm DLK4	34.3	45.7	**70.2**	**88.7**
Eu-D14	24.2	49.8	**70.2**	**89.4**
Angiosperm D14	27.9	56.2	**78.1**	**91.3**
DLK23	4.9	20	34.3	60.4
Gymnosperm DLK23	18.1	37.7	63.4	**87.6**
Angiosperm DLK2	10.2	21.2	44.5	68.7
Angiosperm DLK3	21.9	31.7	55.1	**78.1**

Table showing the degrees of protein sequence conservation in various D14/KAI2 clades. Four degrees of conservation were used: ‘invariant’ (>99% of sequences in a given clade have the same amino acid at a given position), ‘highly conserved’ (>90% of sequences in a given clade have the same amino acid at a given position), ‘well conserved’ (>70% of sequences in a given clade have the same amino acid at a given position) and conserved (>50% of sequences in a given clade have the same amino acid at a given position). The values in the table indicate the percentage of positions that fall into these categories in a given clade. So, for instance, 6.8% of positions are invariant in the whole family. Values are cumulative, so ‘conserved’ includes all the positions that are well conserved, highly conserved and invariant. Bold text indicates values greater than 70%

### Eu-KAI2 clade members have strong sequence conservation

Using this approach, we tested the hypothesis that evolution in the *eu-KAI2* super-clade has generally been conservative. We analysed amino acid frequencies from 127 eu-KAI2 proteins and found that 22% of positions are invariant among eu-KAI2 proteins and 89% are conserved (Fig. 4, Table 2). By comparison, in the DDK super-clade only 5.6% of positions are invariant, with 63% conserved (Table 2). Indeed, the level of conservation across eu-KAI2 proteins as a whole is very comparable to conservation within taxon-level KAI2 clades. For instance, the angiosperm eu-KAI2 clade has 24% invariant positions and 94% conserved (Table 2). Together with the short branch lengths, the similarity in the level of between-clade and within-clade conservation in the eu-KAI2 super-clade supports the idea of a conservative evolutionary history.

**Fig. 5:**
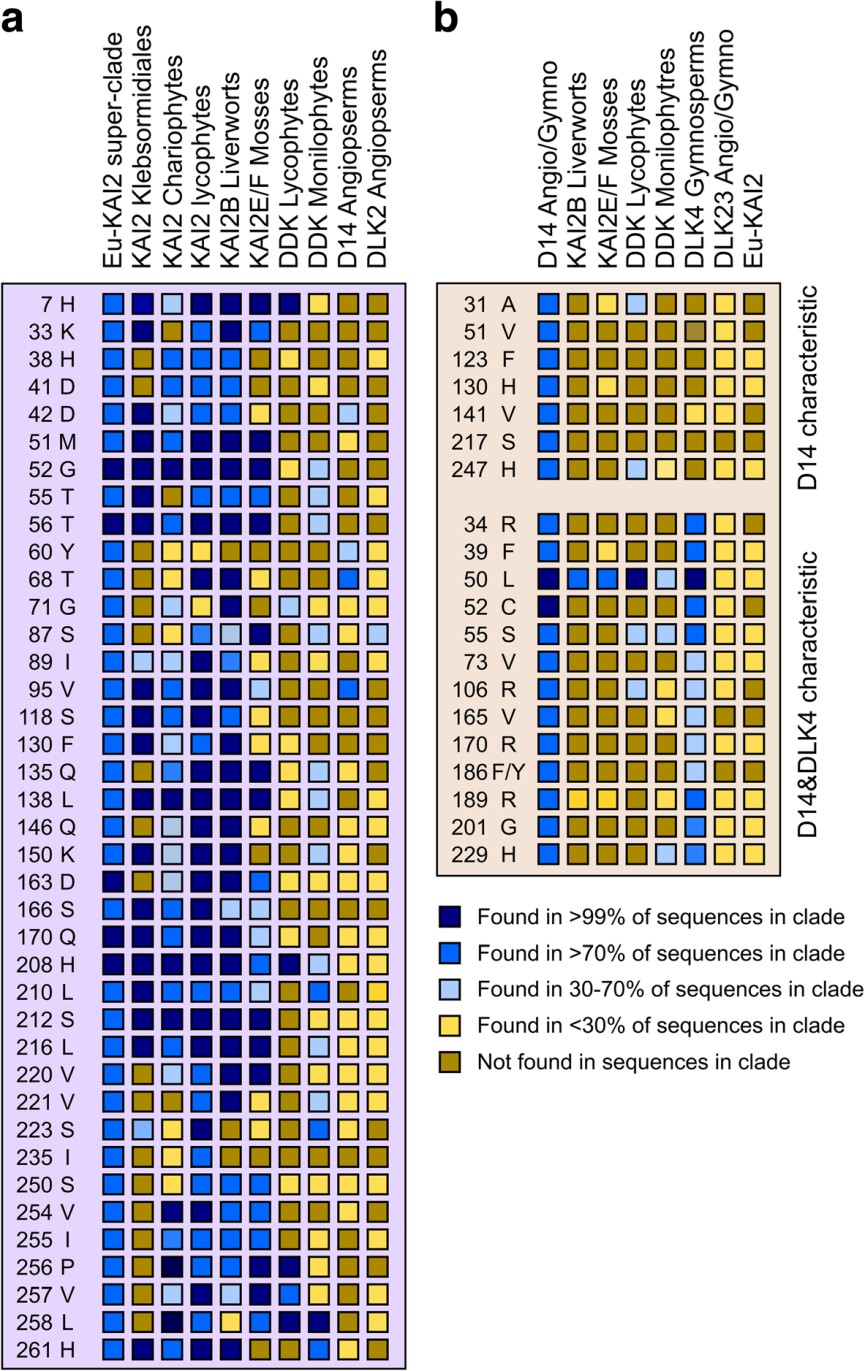
KAI2 and D14 protein characteristics. **a** We identified well-conserved positions in eu-KAI2 proteins (i.e. >70% of sequences have the same amino acid) in which the amino acid is characteristic of eu-KAI2 proteins (i.e. found in <30% of other sequences). These are listed at the *left* (position and amino acid). We then tested whether various clades share elements of this structure (i.e. how frequently the same amino acid is found at the same position in that clade). Charophyte and lycophyte KAI2 proteins are a close match, while KAI2B and KAI2E/F proteins from liverworts and mosses respectively have considerable similarity. However, DDK, D14 and DLK2 proteins do not share these characteristics. **b** We performed the same analysis with eu-D14 proteins, but only identified 7 characteristic residues. We thus extended the search to the combined D14-DLK4 clade and identified another 13 residues characteristic of the wider clade. These are listed at the *left* (position and amino acid). Very little conservation of these characteristic residues is found in other members of the DDK family

Our dataset also allowed us to define a set of residues that are characteristic of eu-KAI2 proteins. We identified 39 positions where the same amino acid is present in at least 70% of eu-KAI2 sequences, and at which the same amino acid is present in less than 30% of DDK clade proteins (Fig. 5a). These are not necessarily the best-conserved positions in eu-KAI2 proteins (Fig. 4), but are those which are most characteristic of eu-KAI2 sequences. When compared to this reference set of residues, individual eu-KAI2 sequences from across the super-clade match at 35–38 out of 39 positions. Conversely, individual D14 sequences only match at 2–5 of these positions, for instance (Fig. 5a, Additional file 8). Eu-KAI2 proteins have therefore been generally well conserved through land plant evolution, which in turn implies conservation of eu-KAI2 function.

### Charophyte D14/KAI2 family members may encode proto-KAI2 proteins

**Fig. 6:**
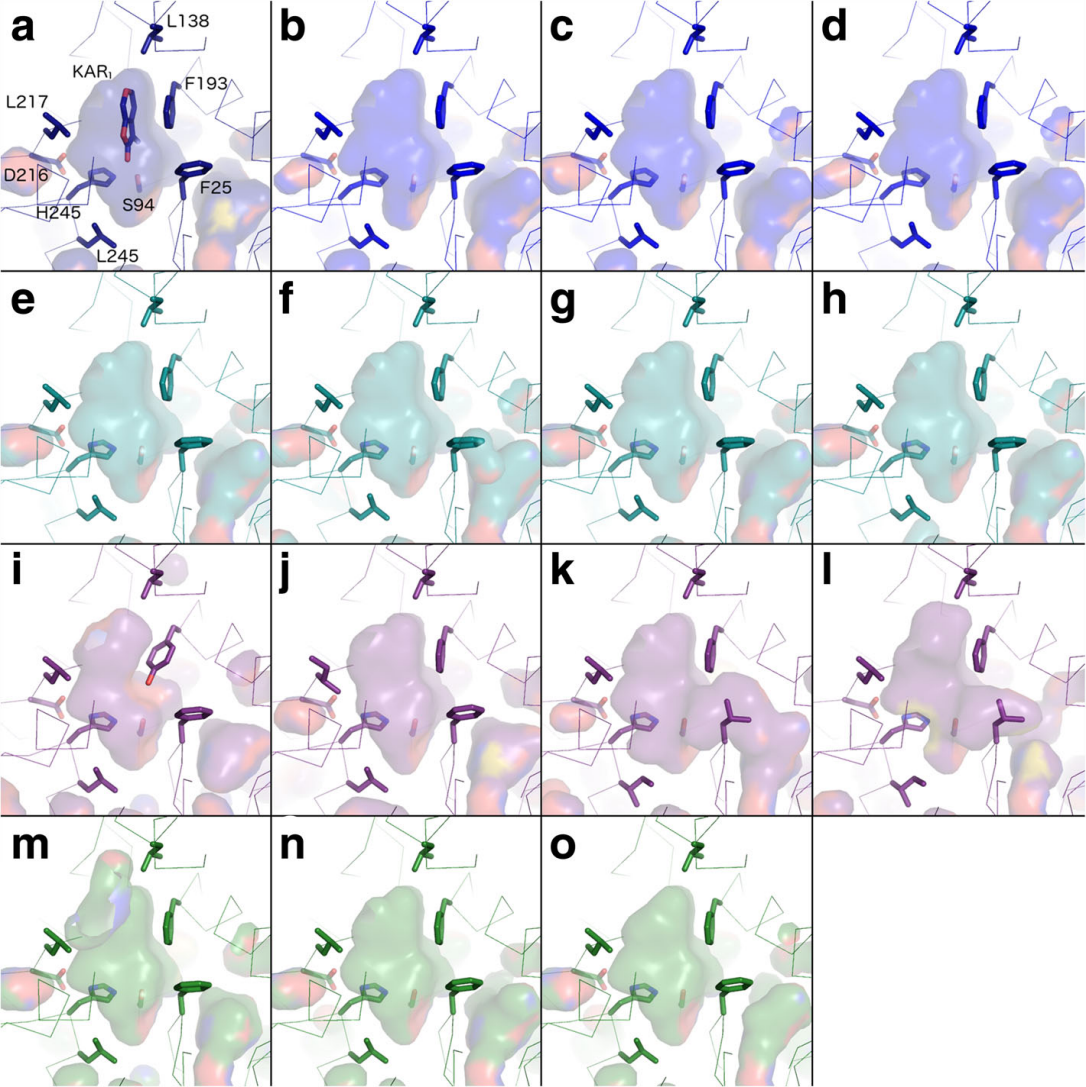
Homology models of KAI2 sequences. Models are shown in *ribbon representation* with the residues that influence the active site cavity shown in *stick representation*. Cavities are depicted as a *transparent surface*. Oxygen, nitrogen and sulphur atoms are coloured *red*, *blue* and *yellow* respectively. **a** The crystal structure of *Arabidopsis thaliana* KAI2 in complex with karrikin (*KAR*_*1*_) is shown in *navy blue* (PDB code 4JYM). Residue numbers correspond to the unified numbering scheme as in Fig. 5; they are –1 relative to those of *A. thaliana* KAI2. **b**–**d** Liverwort KAI2A homology models are shown in *royal blue*; **b** Lejeuneaceae sp. **c** *Lunularia cruciata*, **d** *Ptilidium pulcherrimum*. **e**–**h** Liverwort KAI2B models are shown in *turquoise*; **e** *Riccia berychiana*, **f** *Calypogeia fissa*, **g** *Lunularia cruciata*, **h** *Marchantia polymorpha*. **i**–**l** Charophyte KAI2 models are shown in *purple*; **i** *Klebsormidium subtile*, **j** *Chara vulgaris*, **k** *Coleochaete scutata*, **l** *Coleochaete irregularis*. **m**–**o** Moss KAI2E/F models are shown in *green*; **m** *Sphagnum recurvatum* KAI2E, **n** *Timmia austriaca* KAI2F*,* **o** *Tetraphis pellucida* KAI2F

We examined the charophyte KAI2-like proteins relative to our eu-KAI2 reference set and found that they matched at 20–29 positions (Fig. 5a; Additional file 8). This suggests that while these proteins have relatively strong similarity with eu-KAI2 proteins, they are probably not true KAI2 proteins. To test this idea further, we generated homology models of charophyte KAI2 proteins using the crystal structure of karrikin-bound *Arabidopsis thaliana* KAI2 as a template [50]. Focussing on the ligand binding pocket, we observed that some of the charophyte proteins had pockets similar to that of *A. thaliana* KAI2 (Fig. 6a, i–l; Additional files 9 and 10), while others had larger pockets. This difference is primarily determined by substitution of the ‘intrusive’ phenylalanine residue (F25) that limits the volume of the eu-KAI2 pocket for a leucine residue. These data are consistent with the idea that charophyte KAI2 proteins are similar to eu-KAI2 proteins but do not completely conform to the conserved eu-KAI2 structure.

### Liverwort DDK clade members have conserved KAI2 structure

We next turned our attention to the DDK clade, which has lower overall amino acid conservation. We assessed whether the DDK proteins from liverworts (KAI2B), which have previously been characterized as KAI2-like, have conserved KAI2 features. We found that individual KAI2B proteins match the eu-KAI2 reference set at 29–33 out of 39 positions (Fig. 5a; Additional file 8). Although this is lower than eu-KAI2 proteins from liverworts (KAI2A), it suggests that these proteins could retain aspects of KAI2 primary protein structure. To test this idea, we generated homology models of liverwort KAI2B proteins using the crystal structure of karrikin-bound *Arabidopsis thaliana* KAI2 as a template [50]. In each case, we found that the ligand binding pockets of KAI2B proteins are predicted to be essentially identical to those of Arabidopsis KAI2, and indeed liverwort KAI2A proteins (Fig. 6a–h; Additional files 9 and 10). Thus, while KAI2B proteins may be somewhat divergent relative to eu-KAI2 proteins, they probably still retain key features of eu-KAI2 structure.

### Moss and lycophyte DDK clade members do not have KAI2- or D14-like sequences

Conversely, when we analysed the moss KAI2E and KAI2F proteins, we found that they only matched the KAI2 reference set at 22–24 positions (Fig. 5a, Additional file 8). This is a more considerable divergence from eu-KAI2 than liverwort KAI2B proteins and could imply a corresponding alteration in function. Indeed, structural modelling of the KAI2E/KAI2F proteins from *P. patens* has previously suggested that some of these proteins have altered ligand binding pockets relative to eu-KAI2 proteins in the same species [49]. However, modelling of newly available KAI2E/F sequences from other mosses did not suggest major divergences from the KAI2 binding pocket (Fig. 6m–o).

**Fig. 7:**
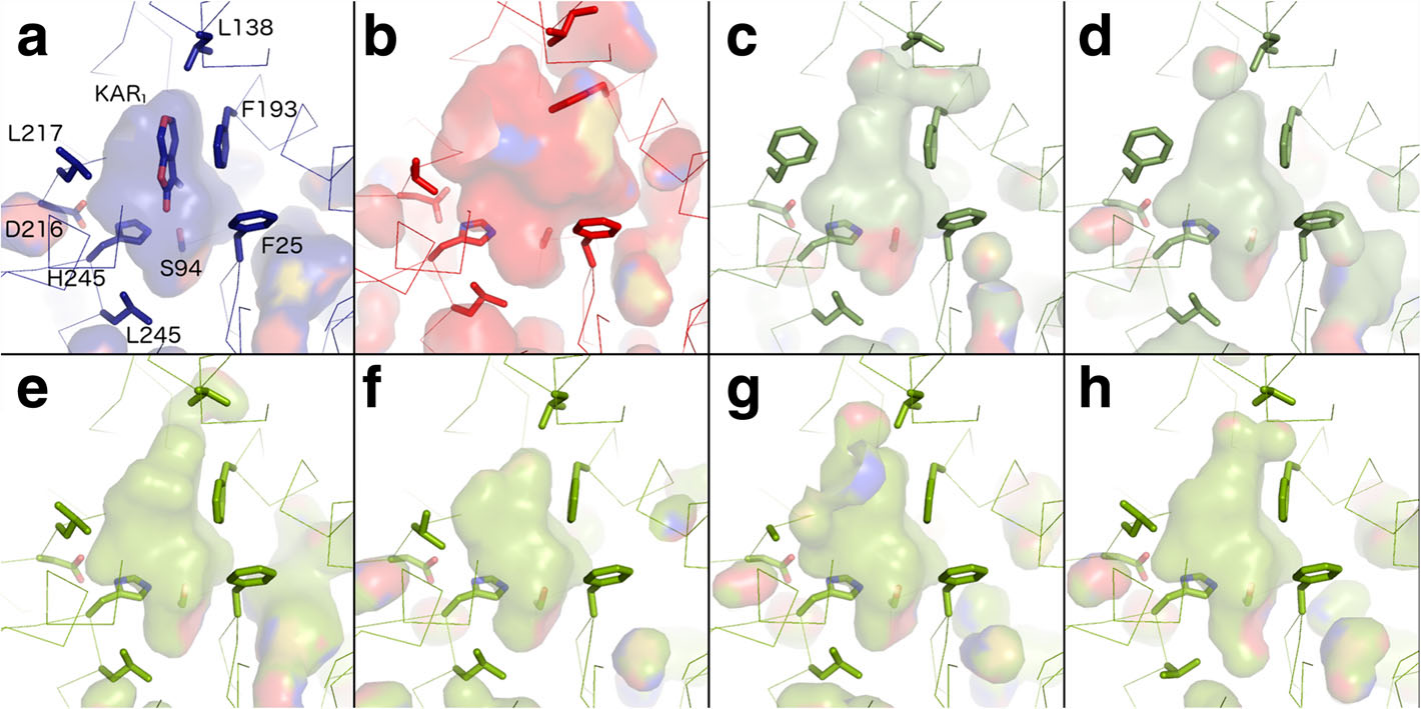
Homology models of DDK sequences. Models are shown in *ribbon representation* with the residues that influence the active site cavity shown in *stick representation*. Cavities are depicted as a *transparent surface*. Oxygen, nitrogen and sulphur atoms are coloured *red*, *blue* and *yellow* respectively. **a** The crystal structure of *A. thaliana* KAI2 in complex with karrikin (*KAR*_*1*_) is shown in *navy blue* (PDB code 4JYM). Residue numbers correspond to the unified numbering scheme as in Fig. 5; they are –1 relative to those of *A. thaliana* KAI2. **b** The crystal structure of *A. thaliana* D14 is shown in *red*. **c, d** Lycophyte DDK homology models are shown in *olive green*; **c** *Selaginella moellendorffii* (previously referred to as KAI2b), **d** *Lycopodium annotinum*. **e**–**h** Monilophyte DDK homology models are shown in *lime green*; **e** *Osmunda* sp. DDKb, **f** *Polypodium amorphum* DDKA, **g** *Cystopteris fragilis* DDKA, **h** *Asplenium platyneuron* DDKB

When we analysed lycophyte DDK proteins, we found that they had much less affinity with eu-KAI2 proteins, matching the reference set at only 5–10 positions (Fig. 5a; Additional file 8). To test whether any of these proteins have signatures of D14-type SL receptors, we tried to identify a reference set of D14-characteristic amino acids comparable to our KAI2 reference set. We identified 13 positions at which the same amino acid is present in more than 70% of proteins in the DLK4/D14 clade, and is found at the same position in less than 30% of sequences in both the eu-KAI2 clade and in the wider DLK23 clade (since none of these proteins are currently considered to be SL receptors) (Fig. 5b). We also identified a further 7 positions with amino acids characteristic of eu-D14 proteins alone (Fig. 5b). Known D14 proteins typically match this reference set at 15–20 out of 20 positions (Additional file 8). When we compared individual KAI2E/F proteins to this reference set, they matched at only 0–4 positions (Fig. 5b; Additional file 8). Similarly, lycophyte DDK proteins only matched the D14 reference set at 1–3 positions. Neither of these types of protein thus displays particular similarity to known strigolactone receptors at the level of primary protein sequence. Furthermore, lycophyte DDK proteins display little specific similarity to any characterized member of the D14/KAI2 family. Consistent with this, homology models of lycophyte DDK proteins predicted ligand binding pockets that were neither KAI2-like nor D14-like (Fig. 7a–d; Additional file 9, Additional file 10).

### Seed plant DLK23 and monilophyte DDK proteins may function independently of MAX2

Recent work has delineated the residues in D14 that are needed for interaction with MAX2-class F-box proteins [9]. We confirm that these 18 residues are strongly conserved in D14 proteins, as suggested by [9]. We also noted that 16 of those residues are very highly conserved in the eu-KAI2 super-clade, strongly suggesting that KAI2 proteins interact with MAX2 proteins through exactly the same interface as D14 (Additional file 11). However, the level of conservation across the D14/KAI2 family as a whole is considerably lower than in either the D14 or KAI2 groups, and we thus examined conservation of MAX2-interaction positions in other clades. Remarkably, we observed that of these 18 positions, 12 were not conserved in the highly divergent monilophyte DDKA and DDKB clades; 6 of these positions were not conserved in any monilophyte DDK protein (Additional file 11). Similarly, we found that 5, 7 and 8 of these positions are not conserved in DLK23, DLK2 and DLK3 proteins respectively (Additional file 11). Curiously, 5 of these positions are not conserved in the DLK4B, despite the MAX2 interface being otherwise conserved in the wider D14/DLK4 clade.

The monilophyte proteins occupy an intermediate position in the DDK clade, and we had therefore expected they would have protein sequences intermediate between the KAI2-like proteins in liverworts and eu-D14 proteins in seed plants. Individual monilophyte DDK proteins match the KAI2 reference set at 5–13 positions and the D14 reference set at 0–5 positions (Fig. 5), suggesting that, like lycophyte DDK proteins, they are not especially similar to characterized proteins, and have unique structural features. Indeed, homology modelling suggests that these proteins have quite variable ligand binding pockets that are generally larger than eu-KAI2 proteins but smaller than D14 proteins (Fig. 7e–h, Additional files 9 and 10). This is consistent with the general level of variation among monilophyte DDK proteins. Similarly, we observed that sequence conservation across the wider DLK23 clade is low; only 5% of positions are invariant, and only 60% conserved (Table 2). As would be expected, none of these proteins show affinity with KAI2 or D14 sequences (Fig. 5). It is therefore possible that loss of MAX2 interaction in these proteins has relaxed the structural requirements for protein function, resulting in divergent sequence characteristics.

### The MAX2 family is highly conserved among land plants and charophyte algae

**Fig. 8:**
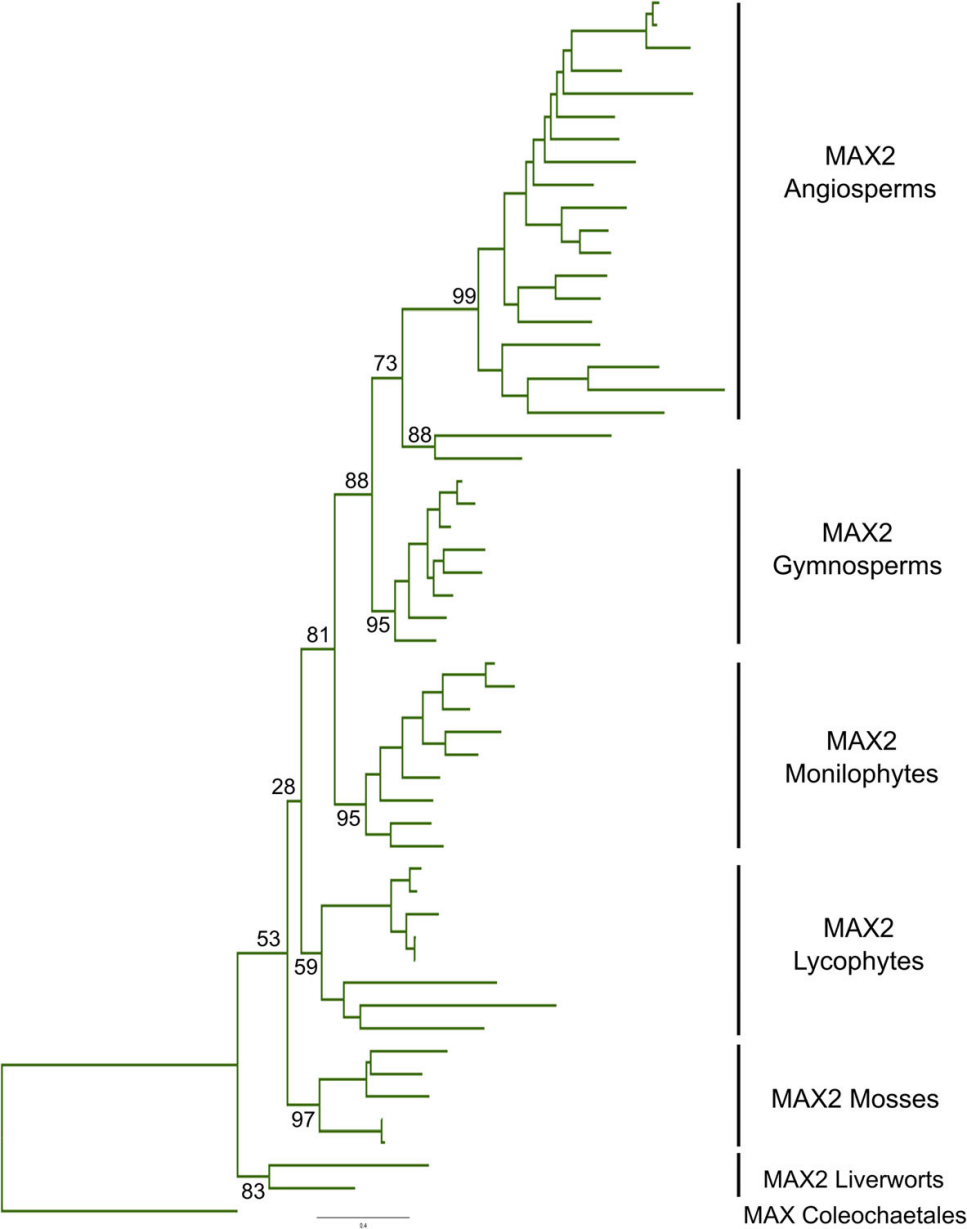
The *MAX2* family has a very conservative evolutionary history. Codon-level phylogenetic analysis implemented in GARLI on the whole *MAX2* family (57 sequences from 54 species). This analysis was performed using an optimized character set (see Methods). Phylogram showing the ‘most likely’ tree from GARLI analysis, labelled to show the high-order relationships between the major clades (as described in Table 1).Trees were rooted with charophyte sequences, consistent with contemporary notions of plant organismal phylogeny. Numbers associated with internal branches denote maximum likelihood bootstrap support (percent support)

The strong conservation between KAI2 and D14 proteins, which are both known to signal through MAX2, strongly implies that the amino acid composition of the MAX2 interface is critical. Furthermore, the strong conservation of the MAX2 interface within the eu-KAI2 clade strongly implies that the cognate interaction surface on MAX2 proteins has not significantly altered throughout the evolution of the land plants. Thus, the lack of conservation in the MAX2 interface in monilophyte DDK proteins, seed plant DLK23 proteins and gymnosperm DLK4B proteins suggests that these groups of proteins may function independently of MAX2. An alternative possibility is that there are additional MAX2 proteins in vascular plants, with an altered cognate interface permitting interaction with these non-conventional DDK super-family proteins. To assess this possibility, we performed a phylogenetic analysis of the *MAX2* family. We obtained 57 sequences from 54 species, representing the major lineages of land plants and charophyte algae (summarized in Additional file 12). We very rarely obtained more than a single *MAX2*-like sequence from any species, and in the instances where we did, these clearly arose from recent duplication events (Fig. 8). Our analysis indicates no long-standing duplications in the MAX2 family, with a single MAX2 clade in each major plant group (Fig. 8). Thus, consistent with previous observations, there appears to have been strong selection to retain *MAX2* as a single-copy gene throughout the evolution of land plants [38]. We also identified a highly conserved MAX2-like protein from a *Coleochaete nitellarum*, suggesting an early origin for KAI2-MAX2 interactions (Additional file 12). However, we did not obtain an obvious *MAX2*-like gene from the completed genome of *Klebsormidium flaccidum*. It was previously suggested that MAX2-like sequences were present in *K. flaccidum* (based primarily on BLAST retrieval rather than protein similarity per se), but re-analysis of these sequences shows that they are only very weakly similar to MAX2-like sequences [51]. Thus, our results suggest that, although there are proto-KAI2 proteins in the Klebsormidiales, these may also signal independently of MAX2.

## Discussion

### KAI2 signalling is highly conserved

Previous studies showed that proteins resembling KAI2 are found throughout land plants and in charophyte algae [23, 30, 34]. Consistent with this, we demonstrate that one of the two major clades in the land plant *D14*/*KAI2* family contains only sequences that strongly resemble Arabidopsis KAI2. We demonstrate with very high resolution that these eu-KAI2 proteins are exceptionally conserved in protein sequence across the clade. Eu-KAI2 proteins have a clearly definable primary protein structure that is distinct from other members of the D14/KAI2 family, and their high levels of conservation arise from both shared-ancestral and shared-derived characteristics (Figs. 5 and 6). These data strongly suggest that there are very specific structural requirements for KAI2 function, and that these functional characteristics have been conserved throughout land plant evolution. Our results demonstrate that D14/KAI2 family proteins from charophytes do not quite meet the definition of eu-KAI2 proteins, but that they do have significant similarity with KAI2 proteins; we have thus categorized them as proto-KAI2. While the function and role of D14 in SL signalling are well understood, KAI2 proteins represent an enigma. In Arabidopsis, KAI2 is required for perception of karrikins, but has clearly defined developmental roles that are unrelated to karrikins; nor is Arabidopsis a naturally fire-following species [21, 23]. This has led to the hypothesis that KAI2 regulates development in response to an unknown endogenous ligand (KL), which is mimicked by karrikins [24, 25]. Consistent with an ancestral role of KL perception, expression of the eu-KAI2 protein from *Selaginella moellendorffii* (SmKAI2A) can partially rescue an Arabidopsis *kai2* mutant but does not restore perception of karrikins [34]. Identification of KL itself will be an important step in understanding the conserved function of KAI2 signalling across land plants [52].

### An early origin for strigolactone signalling?

Previous analyses of the *D14*/*KAI2* family have suggested that the origin of D14-type SL receptors is relatively recent, occurring within the vascular plant lineage, and perhaps restricted to seed plants [23, 30, 34]. Since SL sensitivity seems to be a widespread phenomenon in land plants and perhaps charophytes, this has led to significant speculation that non-canonical SL perception mechanisms exist in non-vascular plants [22, 33]. For instance, it has been suggested that KAI2 proteins could act as SL receptors in mosses and liverworts [22]. Our analyses show that, as far as a distinct primary protein structure can be defined for eu-D14, such proteins do indeed only exist in seed plants. However, the separation of the *DDK* clade (of which eu-D14 proteins are members) from the *eu-KAI2* clade occurred much earlier than previously suspected, at the base of the land plants. This raises the possibility that SL receptors might be a much earlier innovation in the D14/KAI2 family than previously suspected. The DDK protein from *Selaginella moellendorffii* (previously referred to as KAI2b) can hydrolyze SL-like stereoisomers of *rac*-GR24 [34], suggesting that it acts as an SL receptor. We show here that DDK proteins from lycophytes have little specific similarity to D14, which in turn suggests that other proteins in the clade could act as SL receptors despite their non-D14-like structure. However, understanding exactly when SL perception arose in the DDK lineage is contingent on understanding the evolution of land plants themselves. Although the phylogeny of vascular plants is well established, there is still considerable debate regarding the relationship of non-vascular plants, both to each other and to vascular plants. Depending on which scenario is correct, our understanding of the evolution of SL signalling may be considerably altered.

**Fig. 9:**
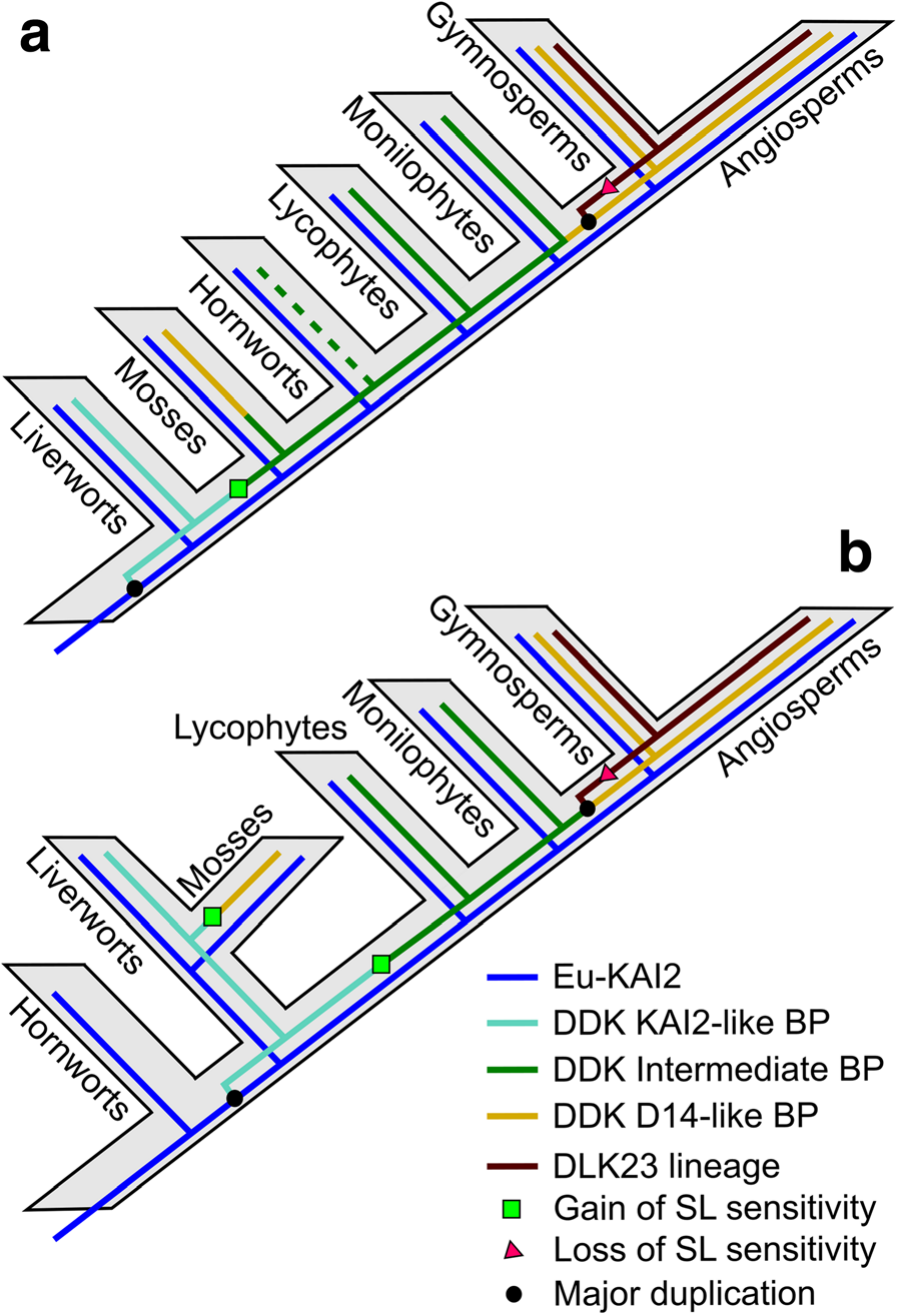
Models of *D14*/*KAI2* evolution. *BP* binding pocket. **a** Traditional model of land plant evolution, with evolution of the *D14*/*KAI2* family superimposed. A single origin of SL perception in the *DDK* lineage would be sufficient to explain known patterns of SL sensitivity. **b** ‘Hornworts-basal’ model of land plant evolution, with evolution of the *D14*/*KAI2* family superimposed. Two independent origins of SL perception in the *DDK* lineage would be required to explain known patterns of SL sensitivity

The ‘traditional’ land plant phylogeny suggests that liverworts, mosses and hornworts form a grade with regard to vascular plants [53]. If this is correct, then the divergence of the eu-*KAI2* and *DDK* lineages would have occurred at the very base of the land plant tree (Fig. 9a). Although slightly divergent in their general structure, liverwort KAI2B proteins appear to have the same ligand binding pockets as eu-KAI2 proteins (Fig. 6). This is consistent with data showing that the KAI2B protein from *Marchantia polymorpha* preferentially hydrolyses non-natural stereoisomers of *rac*-GR24, rather than the SL-like stereoisomers [34]. Indeed, it is currently unclear whether liverworts synthesize or perceive SLs [7]. Under this model of land plant evolution, the evolution of SL perception could be envisaged to have occurred by gradual neo-functionalization of the *DDK* lineage (Fig. 9a). Consistent with this, while KAI2B proteins are structurally similar to eu-KAI2 proteins, the moss proteins in the DDK lineage (KAI2E/F) are more divergent. There is clear evidence for SL perception in *P. patens*, and in this context, it is very interesting to note that a sub-set of *P. patens* D14/KAI2 proteins have previously been predicted to have SL-like ligand binding pockets [49]. All those proteins (KAI2Ea, KAI2Eb, KAI2Fd, KAI2Fe) are members of the *DDK* super-clade in our analysis. However, not all KAI2E/KAI2F proteins from *P. patens* are predicted to have divergent binding pockets [49], and KAI2-like binding pockets were predicted in KAI2E/F proteins from other mosses (Fig. 6). The status of KAI2E/KAI2F proteins as SL receptors is thus far from certain, and more work is needed to firmly establish their structure and function.

A more recent model of land plant evolution suggests that hornworts are the earliest-diverging group of land plants, and that liverworts and mosses form a clade that is sister to vascular plants (Fig. 9b) [31]. The ‘hornworts-basal’ model is controversial, but consistent with it, we only identified a single clade of KAI2-like proteins from hornworts, which in some of our analyses place this clade as a sister clade to all other land plant *D14*/*KAI2* sequences (Figs. 1 and 2). This would suggest that the duplication that created the *eu-KAI2* and *DDK* lineages occurred after the separation of hornworts from all other land plants (Fig. 9b), although it should be noted that the recovery of a single hornwort clade could be due to the limitations of transcriptome databases. The close relationship of liverworts and mosses in this model (irrespective of their placement relative to hornworts) also has major implications for understanding the evolution of SL signalling. If this scenario is correct, then liverwort KAI2B and moss KAI2E/F are probable sister clades. Given the eu-KAI2 like structure of KAI2B protein, this would firmly imply that the ancestral state in the joint KAI2B-E/F clade would involve a KAI2-like binding pocket. If moss KAI2E/F proteins do indeed act as SL receptors, this would mean that SL-like binding pockets would have evolved twice independently in the *DDK* lineage, in mosses and vascular plants (Fig. 9b).

Our ability to precisely understand the origins of SL perception in the *DDK* lineage is thus currently limited by the lack of clarity regarding non-vascular plant phylogeny. It is nevertheless clear that the evolutionary trajectory of the *DDK* lineage is away from an initially KAI2-like structure, and that SL perception probably arose in the lineage at the latest in vascular plants. Given the high conservation between eu-KAI2 proteins, it is therefore very likely that that the majority of proteins in the DDK lineage are at least neo-functional with respect to KAI2. The primary question is thus whether they are neo-functional as SL receptors, or as something rather different. Our data suggest that the structural requirements for SL perception in vascular plants may be relatively relaxed, and even eu-D14 proteins only have limited shared-derived characteristics (Fig. 5a). We speculate that interactions with protein partners (such as SMXL proteins) may have driven the evolution of D14-like structure, rather than requirements for SL perception in itself.

### MAX2-coupled signalling in the D14/KAI2 family

Alongside the origin of specific SL receptors, the evolution of SCF^MAX2^ coupling with D14/KAI2 signalling has also been a subject of debate. Two points have been emphasized; first, that proto-KAI2 proteins are present in charophyte algae, but that MAX2 homologues do not seem to be [30, 38]. Second, *P. patens max2* mutants are reported to have a very different phenotype relative to *P. patens* SL synthesis mutants (no filamentous growth versus excessive filamentous growth), suggesting that they are not in the same pathway [32, 39]. On this basis, it has been suggested that SL signalling in non-vascular land plants might proceed by non-canonical mechanisms [7, 22]. Our data provide us with some insights in this respect. Firstly, the defined MAX2 interface found in D14 is highly conserved across most of the D14/KAI2 family, including in both eu-KAI2 and DDK proteins from liverworts, mosses and hornworts. It therefore seems likely that these proteins do indeed signal via MAX2 in non-vascular plants. We thus hypothesize that the reported *max2* phenotype in *P. patens* arises from a lack of eu-KAI2 signalling, which in turn prevents expression of the SL-deficiency phenotype that would otherwise occur. Furthermore, our data show that the MAX2 interface is also conserved in charophyte D14/KAI2 proteins, tentatively suggesting the existence of MAX2-coupled signalling outside land plants. Consistent with this, we have identified an unambiguous MAX2-like protein in *Coleochaete nitellarum*. In contrast to the strong conservation of D14 and KAI2 proteins, we identified several clades of proteins (DLK2 and DLK3 from angiosperms, DLK23 and DLK4B from gymnosperms, DDKA/DDKB and probably all DDK proteins from monilophytes) that are strongly divergent at the positions that comprise the MAX2 interface. We find no evidence to suggest that these proteins might interact with specialized versions of MAX2. The *MAX2* family has a very strongly conservative evolutionary history (Fig. 8), and there seems to have been a very strong selection pressure to maintain *MAX2* as a single-copy gene. We only identified additional copies of *MAX2* in a few angiosperm genomes known to have recent duplication events (e.g. *Populus trichocarpa*), and we did not identify additional or divergent copies of *MAX2* in any non-angiosperm lineage. Thus, it seems highly likely these DDK proteins with non-conserved MAX2 interfaces signal independently of MAX2.

### A diversity of small molecular receptors?

The DLK23 clade remains the most enigmatic set of proteins in the D14/KAI2 family. Not only do they probably lack the conserved MAX2 interface, but they are highly divergent from other D14/KAI2 proteins and have no known function. DLK2 in Arabidopsis does not seem to be a receptor for SL or KL, at least as far as can be defined genetically [21, 23]. One possibility is that the DLK23 proteins act as receptors for a novel ligand or perhaps multiple ligands. The DLK23 lineage in angiosperms has long internal branches, coupled with a lack of sequence conservation, but there is little evidence of gene loss. This suggests that the high degree of divergence does not simply represent drift in obsolete sequences. Rather, it may indicate continued innovation in the function of DLK23 proteins throughout angiosperm evolution, including the sub- or neo-functionalization process that led to independent DLK2 and DLK3 lineages. Since the DLK23 proteins from early-diverging angiosperms tend to group with eu-DLK2 species in phylogenetic analyses, this tentatively suggests that DLK2 maintained the original structure/function of DLK23, and that the DLK3 lineage is neo-functionalized. In addition to the DLK23 lineage, the fast-evolving DDK super-clade might contain further receptors for non-SL/KL ligands. For instance, since gymnosperms maintain conserved D14-type receptors, it is plausible that DLK4 proteins (and especially the more divergent DLK4B proteins) are not SL receptors. Our work broadens the structural biology platform for D14/KAI2 family members, and future work should provide very interesting insights into the ligand binding, structure and function of these diverse proteins, as well as their interactions with other SL signalling components.

## Conclusions

We conclude that D14-like structure is not required for SL perception, and that SL perception has relatively relaxed structural requirements compared to KAI2-mediated signalling. We propose that SL perception gradually evolved by neo-functionalization within the DDK lineage, and that the transition from KAI2-like to D14-like protein may have been driven by interactions with protein partners, rather than being required for SL perception per se.

## Methods

### Bioinformatic retrieval of *D14*/*KAI2* and *MAX2* sequences

Members of the *D14*/*KAI2* and *MAX2* families were identified by BLAST searches against complete, annotated genomes from two major sources: Phytozome (https://phytozome.jgi.doe.gov/pz/portal.html) or the genome portals for individual species, for instance, the Amborella Genome Project (www.amborella.org). BLAST searches for *D14*/*KAI2* sequences were performed using the full-length coding sequences of *Arabidopsis thaliana D14*, *KAI2* and *DLK2*, using the BLASTN option. BLAST searches for *MAX2* sequences were performed using the highly conserved C-terminal region of *Arabidopsis thaliana MAX2*, using the BLASTN option. Preliminary trees were assembled and used to guide the iterative interrogation of transcriptome databases, particularly those generated by the 1000 Plants (1KP) project (https://db.cngb.org/blast4onekp/). All sequences are listed in Additional file 13. For transcriptome datasets, we BLASTed each major taxonomic group separately. Where novel protein types were identified within a taxon (e.g. Angiosperm DLK3), we re-BLASTed the same taxonomic group with the novel sequence to increase the specificity of our searches. For non-annotated sequences from transcriptome datasets, we searched translations across all six reading frames to identify open reading frames (ORFs), and the longest ORFs were extracted for alignment.

### Alignment

Alignments were initially performed in BioEdit [54] using ClustalW [55]. Full-length sequences from completed genomes were used for the initial alignment, which was manually refined as necessary. We then added sequences from transcriptome databases, many of which are incomplete, but the alignment of full-length sequences provided a scaffold to align these sequences correctly. For *D14*/*KAI2* sequences the resultant alignment of 339 sequences is provided in Additional file 14. For *MAX2* sequences, the resultant alignment of 57 sequences in provided in Additional file 15.

### D14/KAI2 sequence analysis

For primary protein structure analyses, we focussed on positions in the alignment that are present in most sequences. We removed the non-conserved extensions at the N- and C-termini, producing an alignment with 265 core positions. We noted the positions of any non-conserved insertions within this core structure (Fig. 4) and then removed them prior to the final analyses. This 795-nucleotide alignment/265-amino acid alignment was used for analyses of primary protein structure (Figs. 4 and 5, Table 2, Additional file 11). Protein identity comparisons were performed in BioEdit using the ‘protein identity matrix’ function.

### Phylogenetic analysis

For the D14/KAI2 family we performed preliminary phylogenetic analyses to explore the topology of the tree and the effect of inclusion or exclusion of various groups of sequences. We removed 15 nucleotides (5 positions; 57–60, 252) from the 795-nucleotide alignment that were not well conserved across all sequences, leaving a ‘maximum’ phylogenetic alignment of 780 nucleotides. We implemented nucleotide-level maximum likelihood analyses in PhyML [47] and the Genetic Algorithm for Rapid Likelihood Inference (GARLI, v2.0) [46], using the GTR + G + I model of evolution. These analyses are generally congruent with subsequent analyses, but identified some problems with tree reconstruction, particularly with respect to the position of charophyte and lycophyte KAI2 sequences.

For final analyses, the alignment was manually modified in AliView v1.18-beta7 [56], and areas of ambiguous alignment were excluded from subsequent analyses. To determine the optimal model/partitioning scheme, we performed an exhaustive search in PartitionFinder v1.1.1 [57], with each of the three codon positions permitted its own parameters. All models were assessed, branch lengths were constrained to be proportional across partitions and the topology was fixed to that inferred by a preliminary GARLI v2.01 analysis with each codon position given its own GTR + I + G model and rates permitted to vary across partitions; the optimal scheme was selected by the Akaike information criterion (AIC) [58]. Maximum likelihood tree searches were performed under this model (codon positions 1 and 2 with their own GTR + I + G sub-models and codon position 3 with a TVM + I + G sub-model; average rates permitted to vary across partitions) using GARLI v2.01, in the Cyberinfrastructure for Phylogenetic Research (CIPRES) Science Gateway [59]. The GARLI tree searches were performed under the default settings with the exception that genthreshfortopoterm was increased to 40,000; these searches were performed from 48 different random addition sequence starting trees. Support was assessed with 528 bootstrap replicates in GARLI, under the same settings as the best-tree searches, but with each bootstrap search performed from 24 different random addition sequence starting trees. The resulting bootstrap support values were mapped onto our maximum likelihood phylogeny using the SumTrees v3.3.1 program in the DendroPy v3.12.0 package [60].

The MAX2 analyses followed the same workflow, with the exception that the optimal PartitionFinder scheme was for each codon position to have its own GTR + I + G model; searches for the best tree were performed from 10 different random addition starting trees, and 720 bootstrap searches were performed (each from two different random addition starting trees). Three sequences from 1KP accessions known to have contamination issues were pruned from the annotated tree using the Analyses of Phylogenetics and Evolution (APE) package in R [61, 62] prior to the preparation of Fig. 9.

### Assessing tree robustness

We performed multiple analyses to test the robustness of our phylogenetic reconstructions, particularly the placement of KAI2B from liverworts and KAI2E/F from mosses within the DDK clade. Firstly, we removed each DDK clade from the alignment in turn, and re-ran the phylogenetic analysis in PhyML (Additional file 4). The 10 recovered trees have four commonalities: (1) KAI2B is always placed in the eu-KAI2 lineage (except in the ‘No KAI2’ tree), (2) the rest of the DDK clade is always stably grouped together (although there are some variations in the exact branching order within the clade), (3) the relative position of KAI2E/F is completely invariant (except in the ‘No KAI2E/F’ tree and (4) all of the trees place the eu-KAI2 lineage as a grade leading to the DDK clade. This latter point demonstrates that none of these trees are plausible in themselves, since the angiosperm eu-KAI2 clade is placed as a sister clade to the DDK clade containing moss, lycophyte, monilophyte, gymnosperms and angiosperm sequences. Secondly, we ran the analysis on an alignment cut down to match that of [23], using additional RbsQ (bacterial sigma factors with similarity to D14/KAI2 proteins) sequences identified in that study. If we rooted the resulting tree with RbsQ sequences, we observed the same basic topology as in [23]. However, if we rooted with *Selaginella moellendorffii* KAI2, we obtained the same basic topology as in our main analyses, albeit with RbsQ as an in-group in the DDK lineage. Our analysis is thus congruent with the previous analysis in [23].

### Protein homology modelling

KAI2 and DDK sequences were modelled using the SWISSMODEL server (http://swissmodel.expasy.org) [63] based on the ClustalW multiple sequence alignment of KAI2 and DDK sequences as described earlier in this manuscript. No further manipulation of the alignment was performed. Numerous KAI2 crystal structures were available for use as a model template [50, 64–66]; however, we chose the karrikin-bound *A. thaliana* structure (Protein Data Bank (PDB) code 4JYM) [50] as it was the most informative for probing the regions of the protein involved in ligand interaction. Modelled sequences share 37–71% sequence identity with *A. thaliana* KAI2 as computed by BioEdit [54] (Additional file 9). Protein structure and homology model figures were generated with PyMOL [67]. Cavities within homology models were visualized using surface mode on the setting ’Cavities & Pockets (Culled)’ within PyMOL. Volume calculations were performed using the Computed Atlas of Surface Topography of proteins (CASTp) protein server [68] using a probe radius of 1.4 Å. Initial calculations of volume misleadingly included regions of the surface of the protein adjacent to the cavity opening. This problem was circumvented by artificially blocking the cavity opening with a free alanine residue which was not covalently attached to the protein molecule. This alanine was placed in the same *xyz* coordinates for all superposed homology models and crystal structures.

To independently confirm the robustness of the generated homology models, 10 representative sequences were also modelled using the I-TASSER server (http://zhanglab.ccmb.med.umich.edu/I-TASSER/) [69–71]. To confirm that both methods generated similar models, root-mean-square deviation (RMSD) values for the SWISSMODEL- and I-TASSER-generated models were then calculated for the 10 pairs of sequences using the SuperPose server based on the Cα coordinates (http://wishart.biology.ualberta.ca/superpose/) [72]. The RMSD values confirmed that both model-generating servers converged on essentially the same result (Additional file 16).

## Additional files

Additional file 1: Sampling of *D14*/*KAI2* family members. Table showing *D14*/*KAI2* family sampling rates across the plant kingdom. The primary taxonomic divisions are shown at the *left*; lycophytes (*L*), monilophytes (*M*), gymnosperms (*G*) and angiosperms (*A*) are further broken down into major sub-groups. The number of species (*unshaded*) and the number of sequences (*shaded*) obtained from each taxon are shown. Numbers for the Poaceae are shown separately from other Poales, which are in turn shown separately from other commelinids. (DOCX 16 kb)

Additional file 2: Preliminary analysis of D14/KAI2 family phylogeny. Cladogram showing the most likely tree from codon-level phylogenetic analysis on the D14/KAI2 family implemented in GARLI on complete sequence set (339 sequences) using a partially optimized alignment. The tree was rooted with charophyte sequences. *M*-*C*(-*E*) magnoliids, chloranthales, (eudicots). (PNG 498 kb)

Additional file 3: The eu-KAI2 and DDK super-clades diverged early in land plant evolution. Nucleotide-level phylogenetic analysis implemented in PhyML on the D14/KAI2 family, minus charophyte and lycophyte KAI2 sequences (296 sequences). This analysis was performed using the full-length dataset (780 characters). Trees were rooted with liverwort KAI2 sequences. A) Phylogram showing the ‘most likely’ tree from PhyML analysis, labelled to show the high-order relationships between the major clades (as described in Table 2). B) Cladogram depicting the phylogenetic tree from A) in simplified form. Major clades and sub-clades (as listed in Table 2) are collapsed. Numbers associated with internal branches denote maximum likelihood bootstrap support (percent support). (PNG 750 kb)

Additional file 4: Influence of dataset of D14/KAI2 family topology. Cladogram depicting the outcome of maximum likelihood analysis (implemented in PhyML) on an early dataset including protein sequences from multiple complete angiosperm genomes and the completed genomes of *Picea abies* (gymnosperm), *Selaginella moellendorffii* (lycophyte), *Physcomitrella patens* (moss) and *Klebsormidium flaccidum*, plus an expressed sequence tag (*EST*) sequence from *Coleochaete nitellarum* (102 sequences, 259 characters). The topology is congruent with that obtained from the larger, final dataset. (JPG 694 kb)

Additional file 5: The eu-KAI2 and DDK super-clades diverged early in land plant evolution. Cladogram depicting the phylogenetic tree from Fig. 1, but rooted with KAI2 sequences from hornworts to show the effect of root choice on the relative arrangement of basal clades. (PNG 413 kb)

Additional file 6: Testing the robustness of the D14/KAI2 reconstruction. Cladograms depicting the outcome of maximum likelihood analysis (implemented in PhyML) on the D14/KAI2 dataset (minus charophyte and lycophyte KAI2 sequences) when each DDK clade is removed in turn (as indicated below the tree). All trees were rooted with liverwort KAI2A sequences. The 10 trees are fundamentally similar, placing all eu-KAI2 sequences (coloured *dark blue*) + liverwort KAI2B (indicated by *) as a grade relative to the remainder of the DDK clade (coloured as in Fig. 1). The branching order within the remainder of the DDK clade is relatively stable, although some clades swap positions, here indicated by a *double-headed arrow*. Most often gymnosperm DLK4 becomes sister to gymnosperm D14 rather than angiosperm D14, or the lycophyte and monilophyte DDK sequences swap positions. (PNG 996 kb)

Additional file 7: Comparison to previous phylogenies. A) Phylogram depicting the outcome of amino acid level maximum likelihood analysis (implemented in PhyML) on a sequence dataset pruned to match that of Waters et al. [23], with the inclusion of three RbsQ sequences from bacteria (55 sequences, 260 characters). Tree rooted with RbsQ sequences. This tree is very similar to that depicted in Waters et al. [23]. B) The same tree as in A), but rooted with the *Selaginella moellendorffii* KAI2 sequence. The tree displays the same general topology as our main phylogenetic reconstructions, albeit with RbsQ as an in-group in the DDK lineage. Out analyses are therefore essentially congruent with that of Waters et al. (PNG 861 kb)

Additional file 8: Comparisons to KAI2/D14 reference amino acid sets. Excel file listing the reference KAI2-specific and D14-specific sets of amino acids (see Fig. 5) and showing the match between individual sequences and these reference sets. (XLSX 41 kb)

Additional file 9: Cavity volumes of KAI2 and D14 crystal structures and homology models. Cavity volumes of KAI2 (Protein Data Bank (*PDB*) codes 4JYM, 4JYP (Guo et al. [50]), 5DNU, 5DNV (Xu et al. [73])) and D14 (PDB codes 4DNP (Hamiaux et al. [12]), 4IH4 (Zhou et al. [17]), 3WIO (Nakamura et al. [14])) and homology models were calculated using the Computed Atlas of Surface Topography of proteins (*CASTp*) server (Dundas et al. [68]). (DOCX 20 kb)

Additional file 10: Homology models of D14/KAI12 proteins. Models are shown in *ribbon representation* with the residues that influence the active site cavity shown in *stick representation*. Cavities are depicted as a *transparent surface*. Oxygen, nitrogen and sulphur atoms are coloured *red*, *blue* and *yellow* respectively. (A–D) Liverwort KAI2A homology models are shown in *royal blue*; (A) *Bazzania trilobata*, (B) *Marchantia paleacea*, (C) *Marchantia polymorpha*, (D) *Riccia berychiana*. (E–H) Charophyte KAI2 models are shown in *purple*; (E) *Cylindrocystis cushleckae*, (F) *Klebsormidium flaccidum*, (G) *Netrium digitus*, (H) *Roya obtusa*. (I, J) Lycophyte DDK models are shown in *olive green*; (I) *Selaginella stauntoniana*, (J) *Huperzia myrsinites*. (K–S) Monilophyte DDK models are shown in *lime green*; (K) *Botrypus virginianus*, (L) *Cyathea spinulosa,* (M) *Hymenophyllum bivalve*, (N) *Sceptridium dissectum,* (O) *Tmesipteris parva*, (P) *Asplenium platyneuron* DDK1*,* (Q) *Vittaria lineata* DDK1, (R) *Cystopteris fragilis* DDK2*,* (S) *Diplazium wichurae* DDK2*. (PNG 2553 kb)*

Additional file 11: MAX2-interacting residues. The residues in the leftmost column are those identified by Yao et al. [9] as playing a role in the interaction of Arabidopsis D14 with D3 (=MAX2) from rice. Numbers in the first column are relative positions with the AtD14 protein; these are corrected to our unified system in the second column. The consensus amino acids at those positions in the whole family, eu-D14 and eu-KAI2 clades are given in the next three columns. Shading indicates the degree of conservation at the position (*pale blue* >50%, *light blue* >70%, *mid-blue* >90%, *dark blue* >99%, *purple* 100%). The final column indicates clades in which these residues are not conserved. (DOCX 17 kb)

Additional file 12: Sampling of MAX2 family members. Table showing MAX2 family sampling rates across the plant kingdom. The primary taxonomic divisions are shown at the *left*; lycophytes (*L*), monilophytes (*M*), gymnosperms (*G*) and angiosperms (*A*) are further broken down into major sub-groups. The number of species (*unshaded*) and the number of sequences (*shaded*) obtained from each taxon are shown. (DOCX 16 kb)

Additional file 13: List of sequences. Excel files listing all the sequences identified in this study, accession numbers and source. (XLSX 30 kb)

Additional file 14: D14/KAI2 alignment. Nexus file containing all *D14*/*KAI2* sequences in alignment. (NEX 271 kb)

Additional file 15: MAX2 alignment. Nexus file containing all *MAX2* sequences in alignment. (NEX 175 kb)

Additional file 16: Comparison of SWISSMODEL- and I-TASSER-generated homology models. Root-mean-square deviation (*RMSD*) values were calculated for 10 representative sequences modelled in I-TASSER compared to their SWISSMODEL counterpart. *The sequence of *A. thaliana* KAI2, for which there are several crystal structures, was also submitted to the I-TASSER server. The I-TASSER model of *A. thaliana* KAI2 was compared with the *A. thaliana* KAI2 crystal structure 4JYM (Guo et al. [50]), the SWISSMODEL template used in this study, as a control. (DOCX 12 kb)
