# Supplementary material for: Evolution of strigolactone receptors by gradual neo-functionalization of KAI2 paralogues
Source: BMC Biol. 2017 Jun 29;15:52. doi: 10.1186/s12915-017-0397-z (PMC5490202; doi:10.1186/s12915-017-0397-z)
Supplement: Supplementary file 1 — Sampling of D14/KAI2 family members. Table showing D14/KAI2 family sampling rates across the plant kingdom. The primary taxonomic divisions are shown at the left; lycophytes (L), monilophytes (M), gymnosperms (G) and angiosperms (A) are further broken down into major sub-groups. The number of species (unshaded) and the number of sequences (shaded) obtained from each taxon are shown. Numbers for the Poaceae are shown separately from other Poales, which are in turn shown separately from other commelinids. (DOCX 16 kb) [file 12915_2017_397_MOESM1_ESM.docx]

| Charophyte algae | | 10 | 15 |
| --- | --- | --- | --- |
| Marchantiophyta | | 8 | 15 |
| Bryophyta | | 9 | 30 |
| Anthocerotophyta | | 4 | 5 |
| **L** | Lycopodiopsida | 9 | 13 |
|  | Isoetopsida  *Total* | 8  *17* | 9  *22* |
| **M** | Psilotopsida | 5 | 9 |
|  | Equisetopsida | 2 | 2 |
|  | Marattiopsida | 2 | 3 |
|  | Polypodiopsida  *Total* | 10  *29* | 28  *42* |
| **G** | Ginkgophyta | 1 | 4 |
|  | Cycadophyta | 4 | 11 |
|  | Gnetophyta | 3 | 6 |
|  | Pinophyta  *Total* | 24  *32* | 48  *69* |
| **A** | Basal angiosperms | 4 | 7 |
|  | Magnoliids | 2 | 6 |
|  | Basal monocots | 7 | 9 |
|  | Commelinids | 4 | 6 |
|  | Poales | 4 | 5 |
|  | Poaceae | 3 | 16 |
|  | Basal eudicots | 5 | 13 |
|  | Asterids | 2 | 10 |
|  | Rosids  *Tota*l | 13  *44* | 69  *141* |
| ***Totals*** | | *143* | *339* |

**Additional File 1: Sampling of *D14*/*KAI2* family members**

Table showing *D14*/*KAI2* family sampling rates across the plant kingdom. The primary taxonomic divisions are shown at the left; lycophytes (L), monilophytes (M), gymnosperms (G) and angiosperms (A) are further broken down into major subgroups. The number of species (unshaded) and the number of sequences (shaded) obtained from each taxon are shown. Numbers for the Poaceae are shown separately from other Poales, which are in turn shown separately from other commelinids.
