# Supplementary figures and images for: Evolution of strigolactone receptors by gradual neo-functionalization of KAI2 paralogues
Source: BMC Biol. 2017 Jun 29;15:52. doi: 10.1186/s12915-017-0397-z (PMC5490202; doi:10.1186/s12915-017-0397-z)

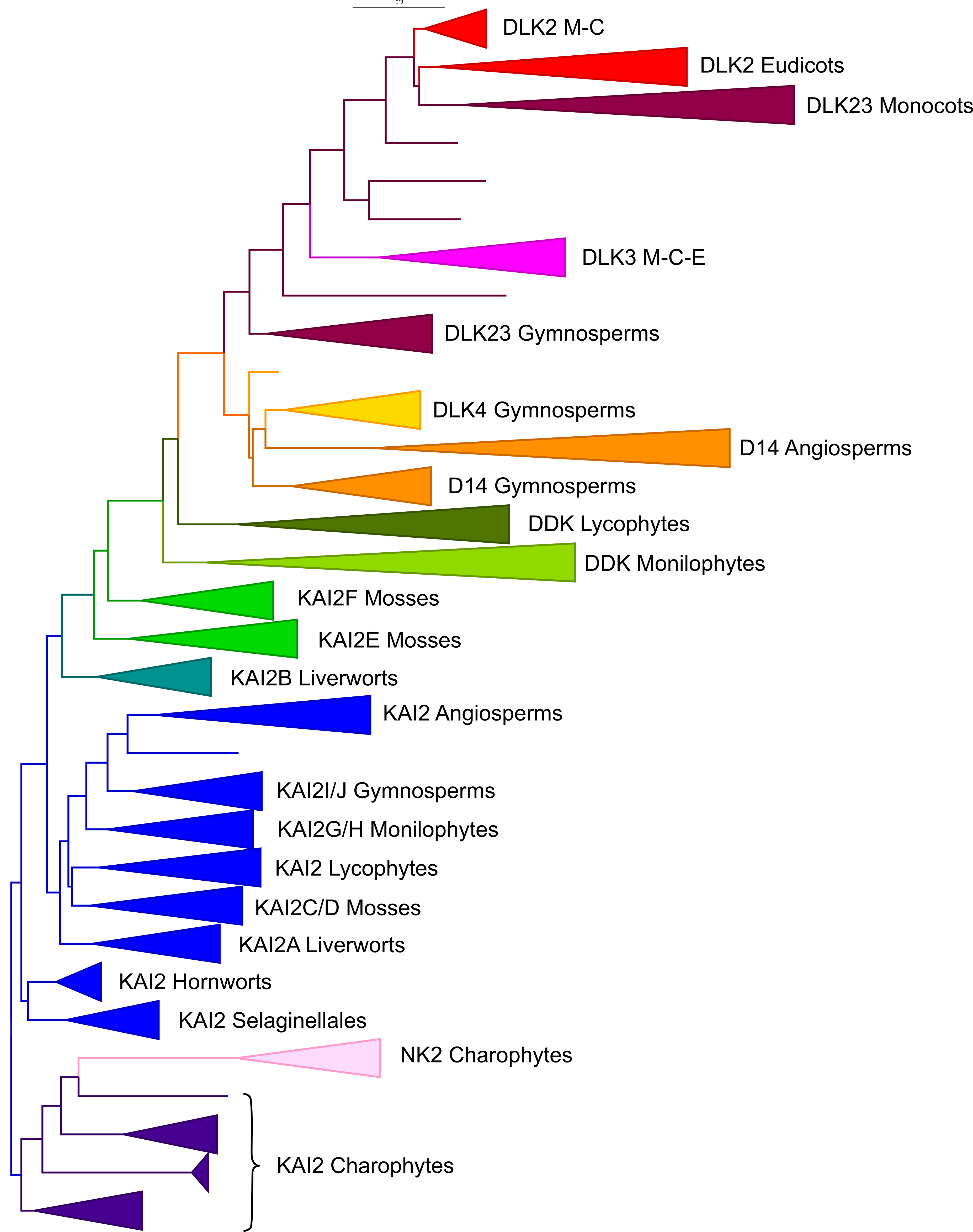

Supplement: Supplementary file 2 — Preliminary analysis of D14/KAI2 family phylogeny. Cladogram showing the most likely tree from codon-level phylogenetic analysis on the D14/KAI2 family implemented in GARLI on complete sequence set (339 sequences) using a partially optimized alignment. The tree was rooted with charophyte sequences. M-C(-E) magnoliids, chloranthales, (eudicots). (PNG 498 kb) [file 12915_2017_397_MOESM2_ESM.png]

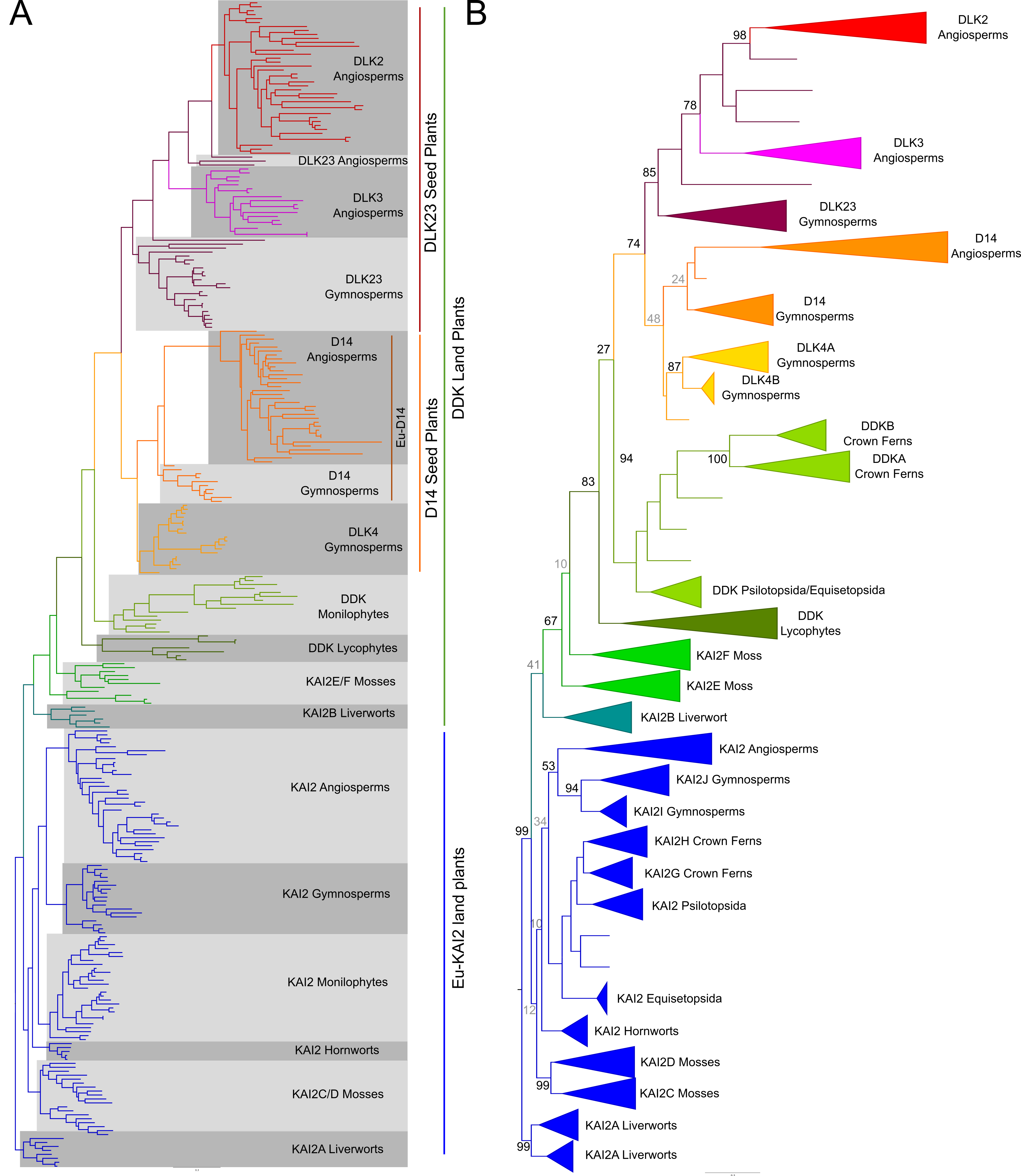

Supplement: Supplementary file 3 — The eu-KAI2 and DDK super-clades diverged early in land plant evolution. Nucleotide-level phylogenetic analysis implemented in PhyML on the D14/KAI2 family, minus charophyte and lycophyte KAI2 sequences (296 sequences). This analysis was performed using the full-length dataset (780 characters). Trees were rooted with liverwort KAI2 sequences. A) Phylogram showing the ‘most likely’ tree from PhyML analysis, labelled to show the high-order relationships between the major clades (as described in Table 2). B) Cladogram depicting the phylogenetic tree from A) in simplified form. Major clades and sub-clades (as listed in Table 2) are collapsed. Numbers associated with internal branches denote maximum likelihood bootstrap support (percent support). (PNG 750 kb) [file 12915_2017_397_MOESM3_ESM.png]

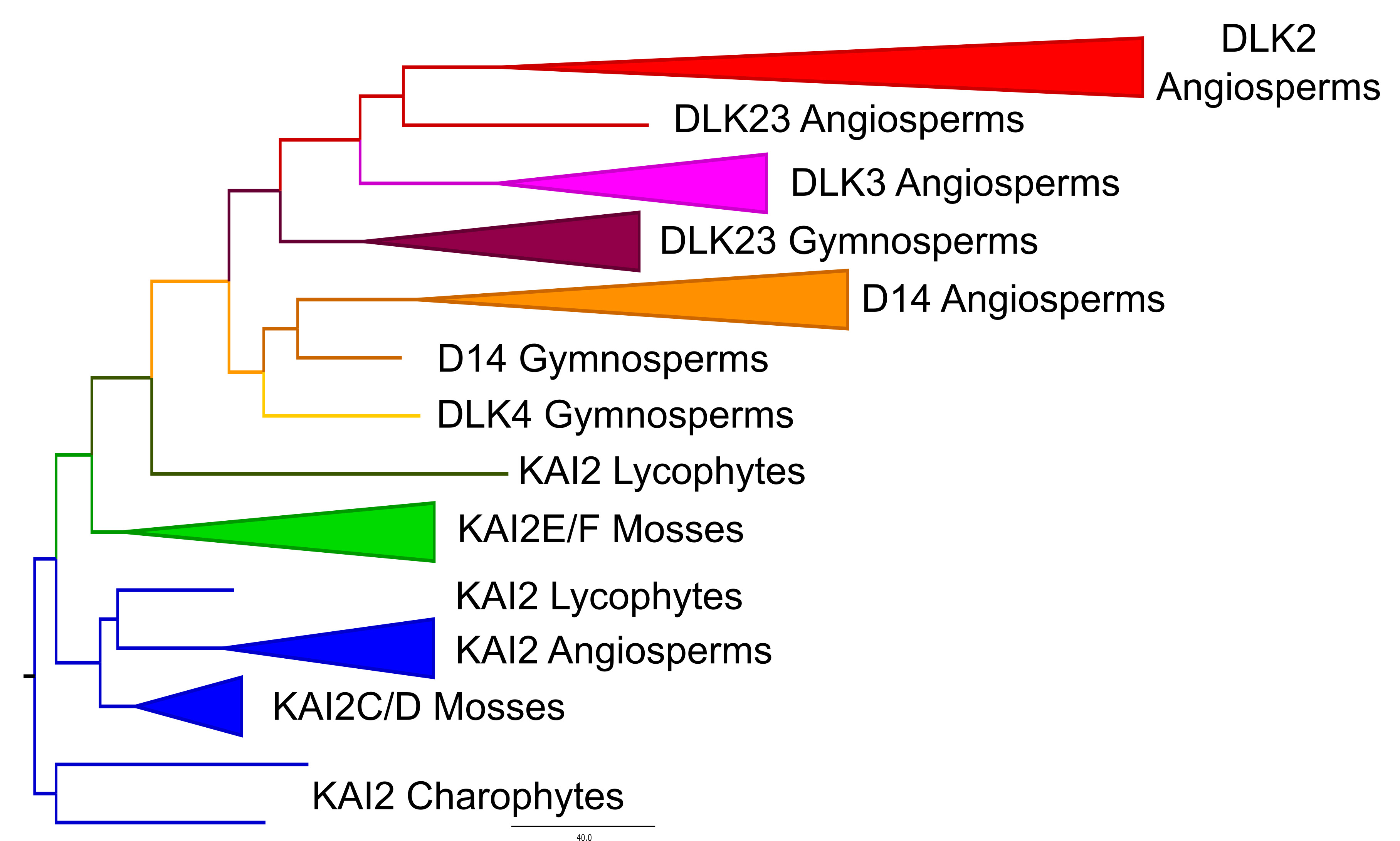

Supplement: Supplementary file 4 — Influence of dataset of D14/KAI2 family topology. Cladogram depicting the outcome of maximum likelihood analysis (implemented in PhyML) on an early dataset including protein sequences from multiple complete angiosperm genomes and the completed genomes of Picea abies (gymnosperm), Selaginella moellendorffii (lycophyte), Physcomitrella patens (moss) and Klebsormidium flaccidum, plus an expressed sequence tag (EST) sequence from Coleochaete nitellarum (102 sequences, 259 characters). The topology is congruent with that obtained from the larger, final dataset. (JPG 694 kb) [file 12915_2017_397_MOESM4_ESM.jpg]

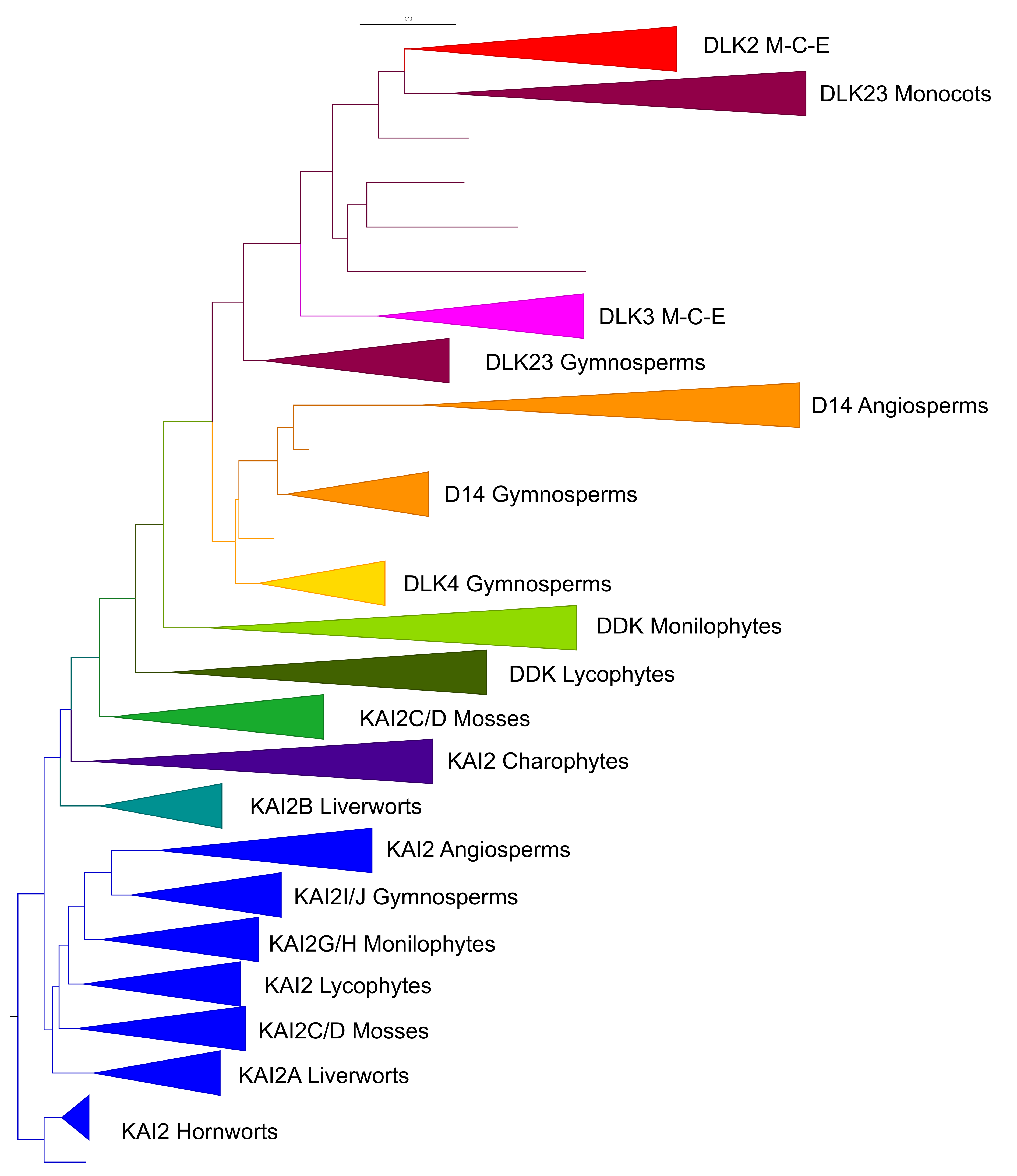

Supplement: Supplementary file 5 — The eu-KAI2 and DDK super-clades diverged early in land plant evolution. Cladogram depicting the phylogenetic tree from Fig. 1, but rooted with KAI2 sequences from hornworts to show the effect of root choice on the relative arrangement of basal clades. (PNG 413 kb) [file 12915_2017_397_MOESM5_ESM.png]

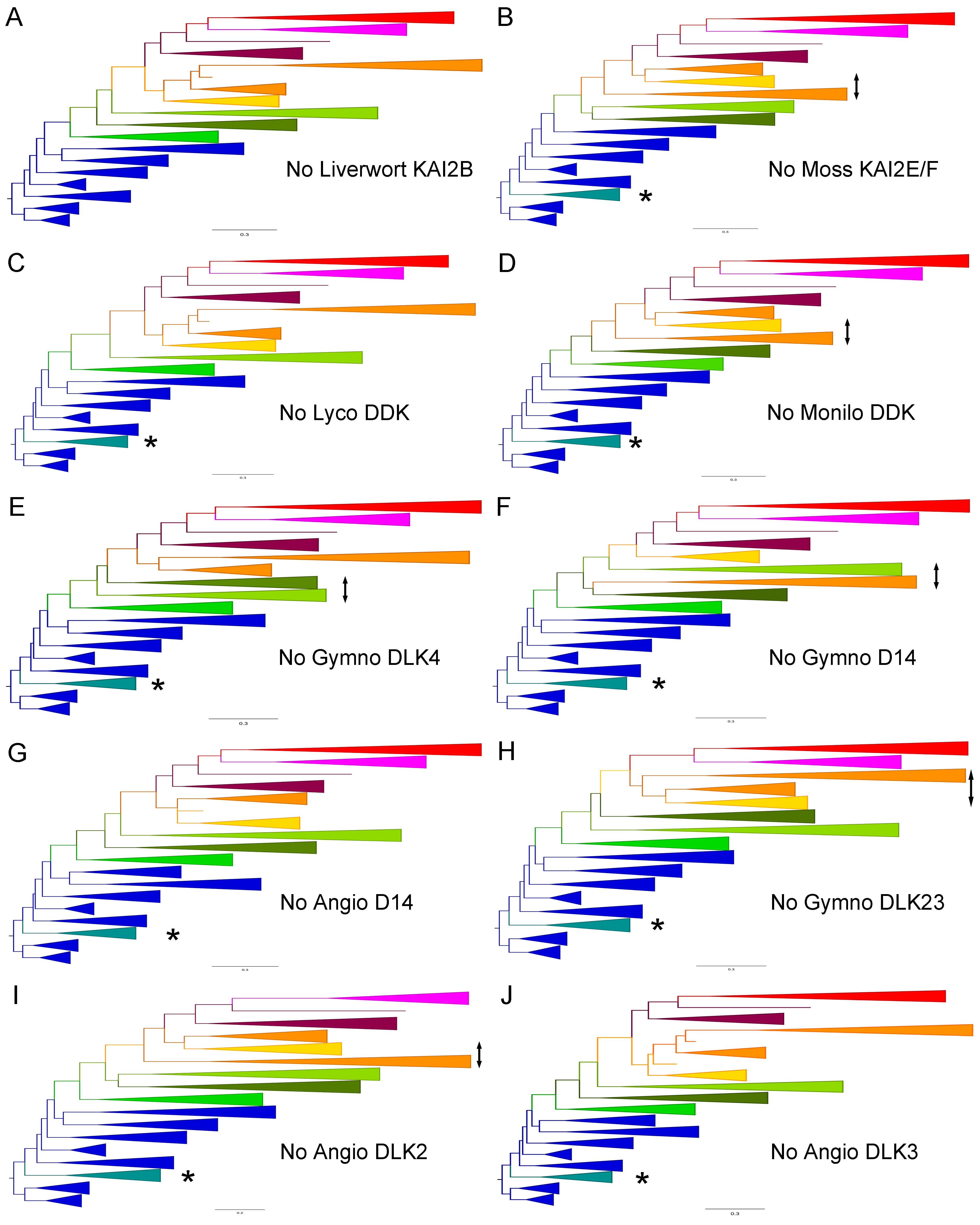

Supplement: Supplementary file 6 — Testing the robustness of the D14/KAI2 reconstruction. Cladograms depicting the outcome of maximum likelihood analysis (implemented in PhyML) on the D14/KAI2 dataset (minus charophyte and lycophyte KAI2 sequences) when each DDK clade is removed in turn (as indicated below the tree). All trees were rooted with liverwort KAI2A sequences. The 10 trees are fundamentally similar, placing all eu-KAI2 sequences (coloured dark blue) + liverwort KAI2B (indicated by *) as a grade relative to the remainder of the DDK clade (coloured as in Fig. 1). The branching order within the remainder of the DDK clade is relatively stable, although some clades swap positions, here indicated by a double-headed arrow. Most often gymnosperm DLK4 becomes sister to gymnosperm D14 rather than angiosperm D14, or the lycophyte and monilophyte DDK sequences swap positions. (PNG 996 kb) [file 12915_2017_397_MOESM6_ESM.png]

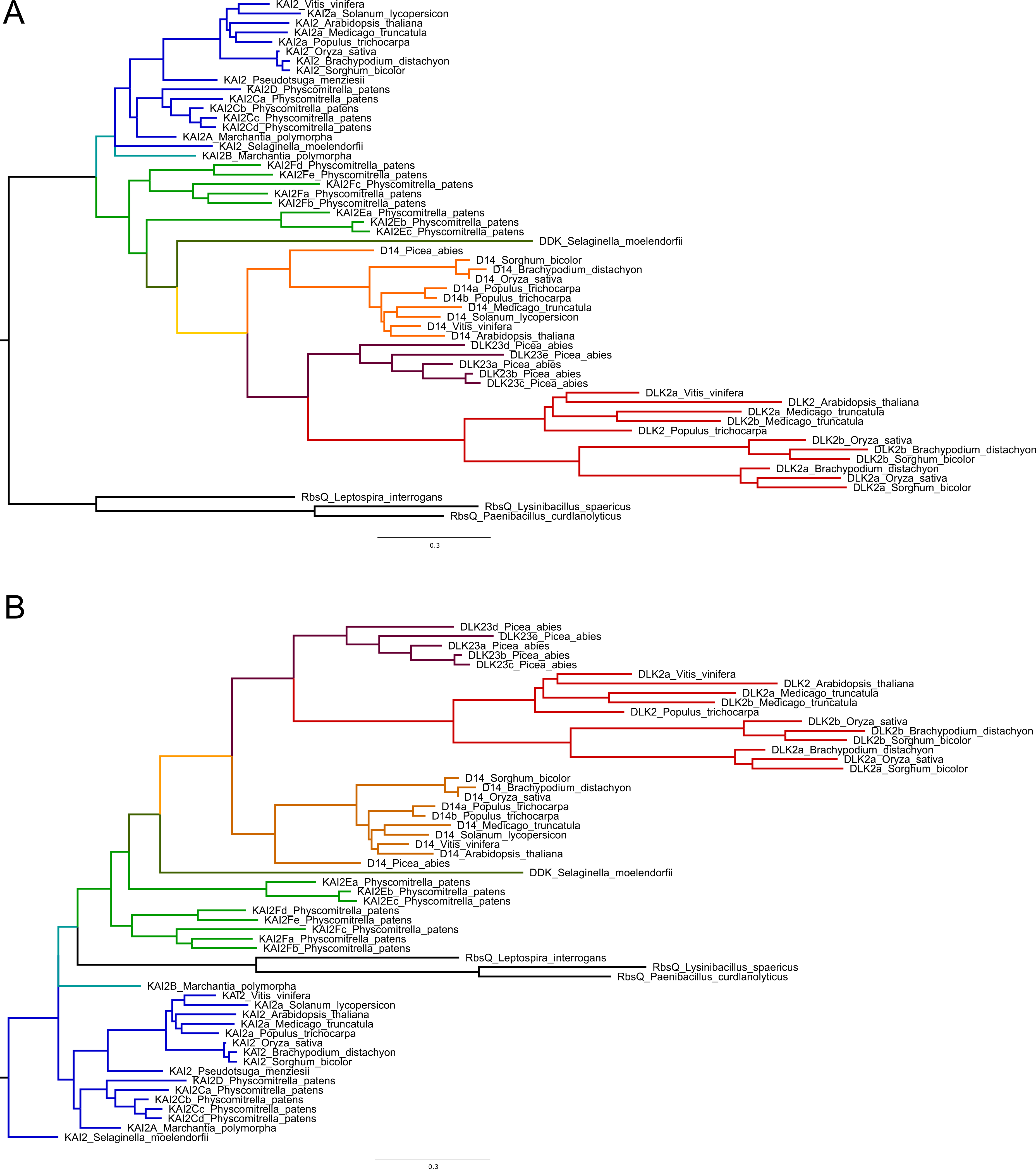

Supplement: Supplementary file 7 — Comparison to previous phylogenies. A) Phylogram depicting the outcome of amino acid level maximum likelihood analysis (implemented in PhyML) on a sequence dataset pruned to match that of Waters et al. [23], with the inclusion of three RbsQ sequences from bacteria (55 sequences, 260 characters). Tree rooted with RbsQ sequences. This tree is very similar to that depicted in Waters et al. [23]. B) The same tree as in A), but rooted with the Selaginella moellendorffii KAI2 sequence. The tree displays the same general topology as our main phylogenetic reconstructions, albeit with RbsQ as an in-group in the DDK lineage. Out analyses are therefore essentially congruent with that of Waters et al. (PNG 861 kb) [file 12915_2017_397_MOESM7_ESM.png]

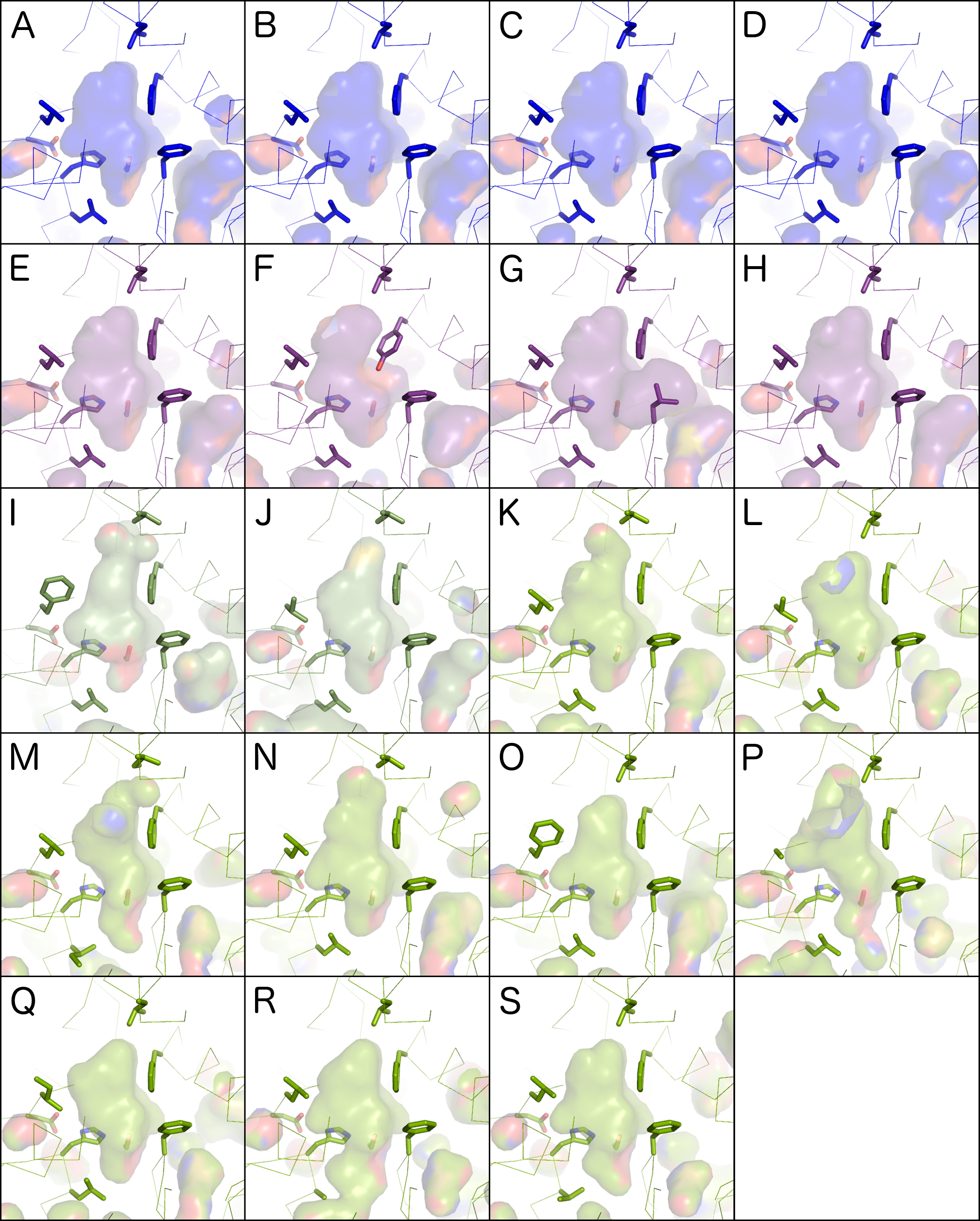

Supplement: Supplementary file 10 — Homology models of D14/KAI12 proteins. Models are shown in ribbon representation with the residues that influence the active site cavity shown in stick representation. Cavities are depicted as a transparent surface. Oxygen, nitrogen and sulphur atoms are coloured red, blue and yellow respectively. (A–D) Liverwort KAI2A homology models are shown in royal blue; (A) Bazzania trilobata, (B) Marchantia paleacea, (C) Marchantia polymorpha, (D) Riccia berychiana. (E–H) Charophyte KAI2 models are shown in purple; (E) Cylindrocystis cushleckae, (F) Klebsormidium flaccidum, (G) Netrium digitus, (H) Roya obtusa. (I, J) Lycophyte DDK models are shown in olive green; (I) Selaginella stauntoniana, (J) Huperzia myrsinites. (K–S) Monilophyte DDK models are shown in lime green; (K) Botrypus virginianus, (L) Cyathea spinulosa, (M) Hymenophyllum bivalve, (N) Sceptridium dissectum, (O) Tmesipteris parva, (P) Asplenium platyneuron DDK1, (Q) Vittaria lineata DDK1, (R) Cystopteris fragilis DDK2, (S) Diplazium wichurae DDK2. (PNG 2553 kb) [file 12915_2017_397_MOESM10_ESM.png]
