# Supplementary material for: Evolution of strigolactone receptors by gradual neo-functionalization of KAI2 paralogues
Source: BMC Biol. 2017 Jun 29;15:52. doi: 10.1186/s12915-017-0397-z (PMC5490202; doi:10.1186/s12915-017-0397-z)
Supplement: Supplementary file 9 — Cavity volumes of KAI2 and D14 crystal structures and homology models. Cavity volumes of KAI2 (Protein Data Bank (PDB) codes 4JYM, 4JYP (Guo et al. [50]), 5DNU, 5DNV (Xu et al. [73])) and D14 (PDB codes 4DNP (Hamiaux et al. [12]), 4IH4 (Zhou et al. [17]), 3WIO (Nakamura et al. [14])) and homology models were calculated using the Computed Atlas of Surface Topography of proteins (CASTp) server (Dundas et al. [68]). (DOCX 20 kb) [file 12915_2017_397_MOESM9_ESM.docx]

| **Crystal structure PDB code** | **Protein** | **% Identity**  **to AtKAI2** | **Pocket volume (Å^3^)** |
| --- | --- | --- | --- |
| 4JYM | *Arabidopsis thaliana* KAI2 (karrikin bound) |  | 352 |
| 4JYP | *Arabidopsis thaliana* KAI2 |  | 396 |
| 5DNU | *Striga hermonthica* KAI2B (karrikin bound) |  | 303 |
| 5DNV | *Striga hermonthica* KAI2B |  | 326 |
| 4IH4 | *Arabidopsis thaliana* D14 |  | 605 |
| 3WIO | *Oryza sativa* D14 (butenolide ring bound) |  | 401 |
| 4DNP | *Petunia x hybrida* DAD2 |  | 547 |
| **Clade** | **Organism protein** |  |  |
| Charophyte | *Chara vulgaris* KAI2 | 55.4 | 312 |
|  | *Coleochaete irregularis* KAI2 | 59.6 | 398 |
|  | *Coleochaete scutata* KAI2 | 55.0 | 574 |
|  | *Cylindrocystis cushleckae* KAI2 | 68.6 | 332 |
|  | *Klebsormidium subtile* KAI2 | 57.3 | 231 |
|  | *Klebsormidium flaccidum* KAI2 | 57.3 | 227 |
|  | *Netrium digitus* KAI2 | 65.2 | 437 |
|  | *Roya obtusa* KAI2 | 66.0 | 331 |
| Liverwort | *Riccia berychiana* KAI2A | 70.5 | 330 |
|  | *Ptilidium pulcherrimum* KAI2A | 69.4 | 317 |
|  | *Marchantia polymorpha* KAI2A | 70.1 | 330 |
|  | *Marchantia paleacea* KAI2A | 70.5 | 330 |
|  | *Lunularia cruciata* KAI2A | 69.8 | 329 |
|  | *Lejeuneaceae sp.* KAI2Aa | 68.3 | 318 |
|  | *Bazzania trilobata* KAI2A | 66.7 | 307 |
|  | *Calypogeia fissa* KAI2B | 66.4 | 320 |
|  | *Lunularia cruciata* KAI2B | 69.0 | 324 |
|  | *Marchantia polymorpha* KAI2B | 70.5 | 325 |
|  | *Riccia berychiana* KAI2B | 69.4 | 325 |
|  | *Sphagnum recurvatum* KAI2E | 60.2 | 330 |
|  | *Timmia austriaca* KAI2F | 60.7 | 327 |
|  | *Tetraphis pellucida* KAI2F | 60.6 | 331 |
| Lycophyte | *Huperzia myrisinites* DDK | 45.6 | 320 |
|  | *Lycopodium annotinum* DDK | 46.7 | 307 |
|  | *Selaginella moelendorfii* DDK | 44.5 | 355 |
|  | *Selaginella stauntoniana* DDK | 44.5 | 338 |
| Fern | *Botrypus virginianus* DDK | 50.1 | 328 |
|  | *Cyathea spinulosa* DDK | 44.1 | 288 |
|  | *Hymenophyllum bivalve* DDK | 48.6 | 326 |
|  | *Sceptridium dissectum* DDK | 50.1 | 339 |
|  | *Tmesipteris parva* DDK | 47.5 | 315 |
|  | *Asplenium platyneuron* DDK1 | 36.9 | 263 |
|  | *Cystopteris fragilis* DDK1 | 42.2 | 292 |
|  | *Polypodium amorphum* DDK1 | 40.7 | 279 |
|  | *Vittaria lineata* DDK1 | 41.8 | 297 |
|  | *Asplenium platyneuron* DDK2 | 43.0 | 333 |
|  | *Cystopteris fragilis* DDK2 | 42.6 | 357 |
|  | *Diplazium wichurae* DDK2 | 45.2 | 319 |
|  | *Osmunda sp.* DDKb | 51.3 | 336 |

**Additional File 9: Cavity volumes of KAI2 and D14 crystal structures and homology models**

Cavity volumes of KAI2 (PDB codes 4JYM, 4JYP (Guo et al, 2013), 5DNU, 5DNV (Xu et al, 2016)) and D14 (PDB codes 4DNP (Hamiaux et al, 2012), 4IH4 (Zhou et al, 2013), 3WIO (Nakamura et al, 2013)) and homology models were calculated using the CASTp server (Dundas et al, 2006).
