# Supplementary material for: Evolution of strigolactone receptors by gradual neo-functionalization of KAI2 paralogues
Source: BMC Biol. 2017 Jun 29;15:52. doi: 10.1186/s12915-017-0397-z (PMC5490202; doi:10.1186/s12915-017-0397-z)
Supplement: Supplementary file 11 — MAX2-interacting residues. The residues in the leftmost column are those identified by Yao et al. [9] as playing a role in the interaction of Arabidopsis D14 with D3 (=MAX2) from rice. Numbers in the first column are relative positions with the AtD14 protein; these are corrected to our unified system in the second column. The consensus amino acids at those positions in the whole family, eu-D14 and eu-KAI2 clades are given in the next three columns. Shading indicates the degree of conservation at the position (pale blue >50%, light blue >70%, mid-blue >90%, dark blue >99%, purple 100%). The final column indicates clades in which these residues are not conserved. (DOCX 17 kb) [file 12915_2017_397_MOESM11_ESM.docx]

| D14 residue | Pos | Consensus | | | Notes | Not conserved in |
| --- | --- | --- | --- | --- | --- | --- |
|  |  | All | Eu-D14 | Eu-KAI2 |  |  |
| N11 | 8 | N | N | N |  |  |
| D31 | 28 | D | D | D |  | DLK2 monocots,  DDKA/DDKB monilo |
| S33 | 30 | S | S | S |  | DLK2 commenelids |
| D52 | 49 | D | D | D |  |  |
| C55 | 52 | G | C | G | C in DLK4/D14  F in DLK23/DLK2/DLK3  G in other proteins |  |
| G57 | 54 | G | G | G |  | DLK3, DD1/DDKB monilo |
| V59 | 56 | T | V | T | V/I in D14/DLK4/DLK23/  DLK2/DLK3  T in other proteins |  |
| G158 | 155 | G | G | G |  | DLK2, DLK3, DLK23,  DD1/DDKB monilo |
| A160 | 157 | A | A | A |  |  |
| P161 | 158 | P | P | P |  | DLK2, DDKA/DDKB monilo |
| L162 | 159 | L | L | L |  | DLK3, DDKA/DDKB monilo |
| A163 | 160 | A | A | A |  | DLK3, DLK23, DDKA/DDKB monilo |
| V164 | 161 | V | V | V |  |  |
| E174 | 171 | E | E | E |  | DLK4B, DLK2, DDK monilo |
| R177 | 174 | R | R | R | Often K instead | DDK monilo |
| F180 | 177 | F | F | F |  | DLK4B, DLK2, DLK3, DLK23  DDK monilo, DDK lyco |
| N181 | 178 | N | N | N |  | DLK4B, DLK2, DLK3, DDK monilo, DDK lyco, KAI2 charo |
| E245 | 242 | E | E | E | E, D, Q or N |  |

**Additional File 11: MAX2-interacting residues**

The residues in the left-most column are those identified by Yao et al (2016) as playing a role in the interaction of Arabidopsis D14 with D3 (=MAX2) from rice. Numbers in the first column are relative positions with the AtD14 protein; these are corrected to our unified system in the second column. The consensus amino acids at those positions in the whole family, eu-D14 and eu-KAI2 clades are given in the next three columns. Shading indicates the degree of conservation at the position (pale blue >50%, light blue >70%, mid-blue >90%, dark blue >99%, purple 100%). The final column indicates clades in which these residues are not conserved.
