# Supplementary material for: Evolution of strigolactone receptors by gradual neo-functionalization of KAI2 paralogues
Source: BMC Biol. 2017 Jun 29;15:52. doi: 10.1186/s12915-017-0397-z (PMC5490202; doi:10.1186/s12915-017-0397-z)
Supplement: Supplementary file 16 — Comparison of SWISSMODEL- and I-TASSER-generated homology models. Root-mean-square deviation (RMSD) values were calculated for 10 representative sequences modelled in I-TASSER compared to their SWISSMODEL counterpart. *The sequence of A. thaliana KAI2, for which there are several crystal structures, was also submitted to the I-TASSER server. The I-TASSER model of A. thaliana KAI2 was compared with the A. thaliana KAI2 crystal structure 4JYM (Guo et al. [50]), the SWISSMODEL template used in this study, as a control. (DOCX 12 kb) [file 12915_2017_397_MOESM16_ESM.docx]

| Protein sequence | RMSD (All Cα atoms) |
| --- | --- |
| KAI2 *Arabidopsis thaliana** | 0.43 |
| DDK *Hymenophyllum bivalve* | 0.69 |
| DDK *Selaginella moelendorfii* | 0.91 |
| DDK *Tmesipteris parva* | 0.91 |
| KAI2 *Klebsomidium flaccidum* | 0.62 |
| KAI2 *Netrium digitus* | 0.65 |
| KAI2A *Marchantia polymorpha* | 0.47 |
| KAI2B *Marchantia polymorpha* | 0.63 |
| KAI2E *Sphagnum recurvatum* | 0.42 |
| KAI2F *Timmia austriaca* | 0.55 |
| DDK *Lycopodium annotinum* | 0.92 |

**Additional File 16: Comparing SWISSMODEL and I-TASSER generated homology models**

Root-mean-square deviation (RMSD) values were calculated for 10 representative sequences modelled in I-TASSER compared to their SWISSMODEL counterpart. *The sequence of *A. thaliana* KAI2, for which there are several crystal structures, was also submitted to the I-TASSER server. The I-TASSER model of *A. thaliana* KAI2 was compared with the *A. thaliana* KAI2 crystal structure 4JYM (Guo et al. 2013), the SWISSMODEL template used in this study, as a control.
